# Activation and Stabilization Strategies of Aluminum Metal Anode Toward High Performance Aqueous Al Metal Batteries

**DOI:** 10.1002/adma.202507164

**Published:** 2025-07-01

**Authors:** Huaming Yu, Xiaofeng Zhang, Yaxin Wang, Meilin Li, Wei Chen, Zhe Hu, Minshen Zhu, Yang Huang

**Affiliations:** ^1^ Advanced Materials Thrust, Function Hub The Hong Kong University of Science and Technology (Guangzhou) Guangzhou Guangdong 511400 China; ^2^ Guangdong Provincial Key Laboratory of New Energy Materials Service Safety, College of Materials Science and Engineering Shenzhen University Shenzhen Guangdong 518055 China; ^3^ Department of Biophysics, Institute of Medical Engineering, School of Basic Medical Sciences, Health Science Center Xi'an Jiaotong University Xi'an Shaanxi 710061 China; ^4^ Research Center for Materials Architectures, and Integration of Nanomembranes (MAIN) TU Chemnitz 09126 Chemnitz Germany; ^5^ Material Systems for Nanoelectronics, TU Chemnitz 09107 Chemnitz Germany

**Keywords:** aluminum anodes, aqueous Al metal batteries, corrosion, electrolytes, surface passivation

## Abstract

Aqueous aluminum metal batteries (AAMBs) have garnered significant attention due to the abundant reserves, low cost, high theoretical capacity, and intrinsic safety of aluminum (Al). However, Al^3+^‐based energy storage technologies remain in their nascent stages, facing a multitude of challenges. One major issue is the poor thermodynamic stability of the aluminum metal anode in aqueous electrolytes, stemming from self‐corrosion, surface passivation, or hydrogen evolution reactions. These parasitic reactions dramatically reduce the reactivity, prevent reversible deposition/dissolution of aluminum, and restrict the electrochemical performance of AAMBs. This review spotlights the critical challenges faced by aluminum metal anodes and aqueous electrolytes. Then, recent progress on activating and stabilizing Al metal anode is summarized and discussed in terms of two aspects, including anode engineering and electrolyte optimization. Ultimately, future designs of high reaction activity of Al metal anode and electrolytes with high reversibility, long lifespan, and high energy density are proposed, which potentially facilitate the development of new generation of Al‐based energy storage batteries.

## Graphical abstract

This review spotlights the critical challenges faced by aluminum metal anodes and aqueous electrolytes. Recent progress on activating and stabilizing Al metal anode is summarized and discussed in terms of two aspects, including anode engineering and electrolyte optimization. Finally, some revelatory insights and possible strategies are provided for the future design of high reaction activity of Al anode and electrolytes.

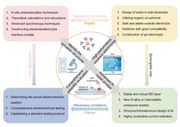

## 1 Introduction

Considering the excessive consumption of traditional fossil fuels which cannot meet the long‐term global energy demands, clean and renewable energy resources (such as solar, wind, and tidal energy) are experiencing exponential growth.^[^ ^1^ ^]^ However, these sustainable energy systems are hindered by their intermittency and uneven spatial and temporal distribution, highlighting the urgent need to develop economical large‐scale energy storage systems (ESS).^[^ ^2^ ^]^ Due to the high energy density, fast response speed, and minimal spatial constraints, lithium‐ion batteries (LIBs) have become the most mature and widely used battery technology in contemporary society since their commercialization in 1991.^[^ ^3^ ^]^ Nevertheless, current LIBs are approaching their theoretical energy density limits, limited lithium resources (only 0.0065% of the earth's crust), and increasingly prominent safety risks that restrict their application in large‐scale ESS.^[^ ^4^ ^]^ Therefore, researchers have devoted significant effort to develop new batteries with other anode materials that are more abundant and cost‐effective than lithium, such as sodium, potassium, calcium, magnesium, zinc, and aluminum.^[^ ^5^ ^]^ These materials are expected to reduce the cost of existing ESS and achieve long‐term sustainability. It is worth noting that the safety of sodium, potassium, magnesium, and calcium metal batteries remains a critical issue due to the flammability and uncontrollability of organic electrolytes, which becomes even more problematic when these batteries are densely arranged in large‐scale ESS.^[^ ^6^ ^]^ Therefore, the development of inherently safe and high‐energy‐density electrochemical large‐scale ESS is an urgent priority.

**Figure 1:**
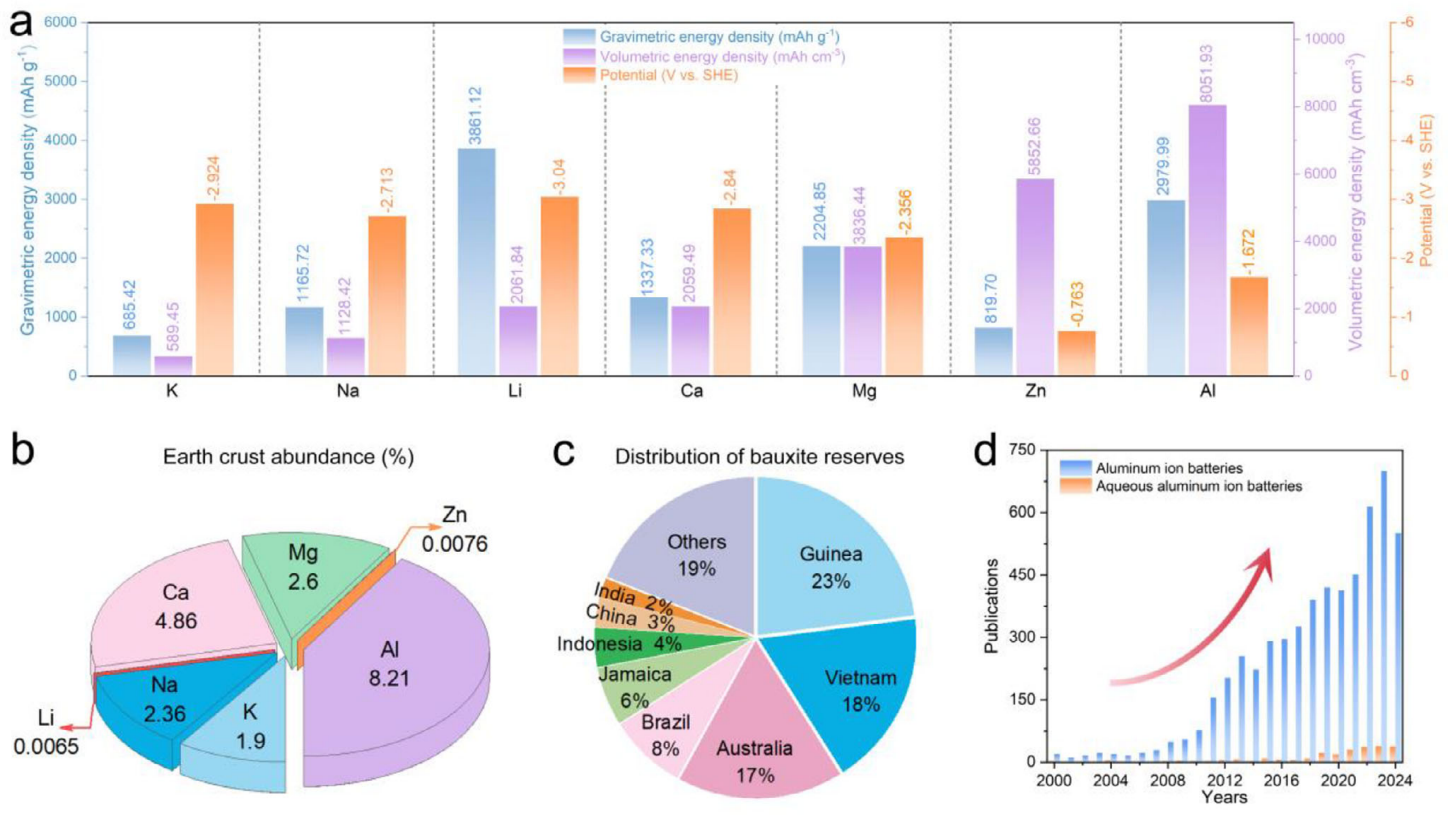
a) Comparison of the energy densities and standard electrochemical reduction potentials of different metal anodes. b) Comparison of the earth's crust abundance of various metals. c) Comparison of the distribution of bauxite reserves. d) The number of publications each year of aluminum ion batteries and aqueous aluminum ion batteries from 2000 to October 2024 from the Web of Science.

Rechargeable batteries operating under aqueous electrolyte conditions are receiving widespread attention due to their inherent safety and low manufacturing costs.^[^ ^7^ ^]^ Additionally, as shown in **Figure** **1**a, among various metal anode materials, the aluminum (Al) anode stands out as one of the most promising energy storage alternatives due to its high theoretical specific capacity, low redox potential (−1.672 V vs standard hydrogen electrode, SHE), and low cost.^[^ ^8^ ^]^ Moreover, aluminum is the most abundant metallic element in the earth's crust (8.2%), which implies lower raw material costs and more sustainable consumption (Figure 1b). Specifically, in 2021, the global reserves of bauxite were approximately 32 billion tons, with the most abundant reserves in countries such as Guinea, Australia, Vietnam, and Brazil (Figure 1c).^[^ ^9^ ^]^ The bauxite reserves of Guinea are about 7.4 billion tons, accounting for ≈23.13% of the reserves of world. In recent years, rechargeable zinc‐ion batteries using neutral or weakly acidic electrolytes have been experiencing a research boom. Compared to aqueous zinc metal batteries, aqueous aluminum metal batteries (AAMBs) have the following advantages: (1) A unique three‐electron transfer mechanism enables aluminum metal to achieve extremely high gravimetric (2979.99 mA h g^−1^ for Al and 819.70 mA h g^−1^ for Zn) and volumetric capacity (8051.93 mAh cm^−3^ for Al and 5852.66 mAh cm^−3^ for Zn). (2) Extremely high crustal abundance (8.21% for Al and 0.0076% for Zn). (3) Lower raw material and manufacturing costs. (4) Lower redox potential (−1.672 V for Al and ‐0.763 V for Zn) and higher operating voltage.^[^ ^10^ ^]^ Noticeably, the price and regeneration cost of aluminum metal is significantly lower than the metals commonly used in other batteries (lithium, sodium, copper, lead, etc.).^[^ ^11^ ^]^ Apparently, AAMBs with aluminum metal anode have become a very promising candidates for the development of advanced ESS.

**Figure 2:**
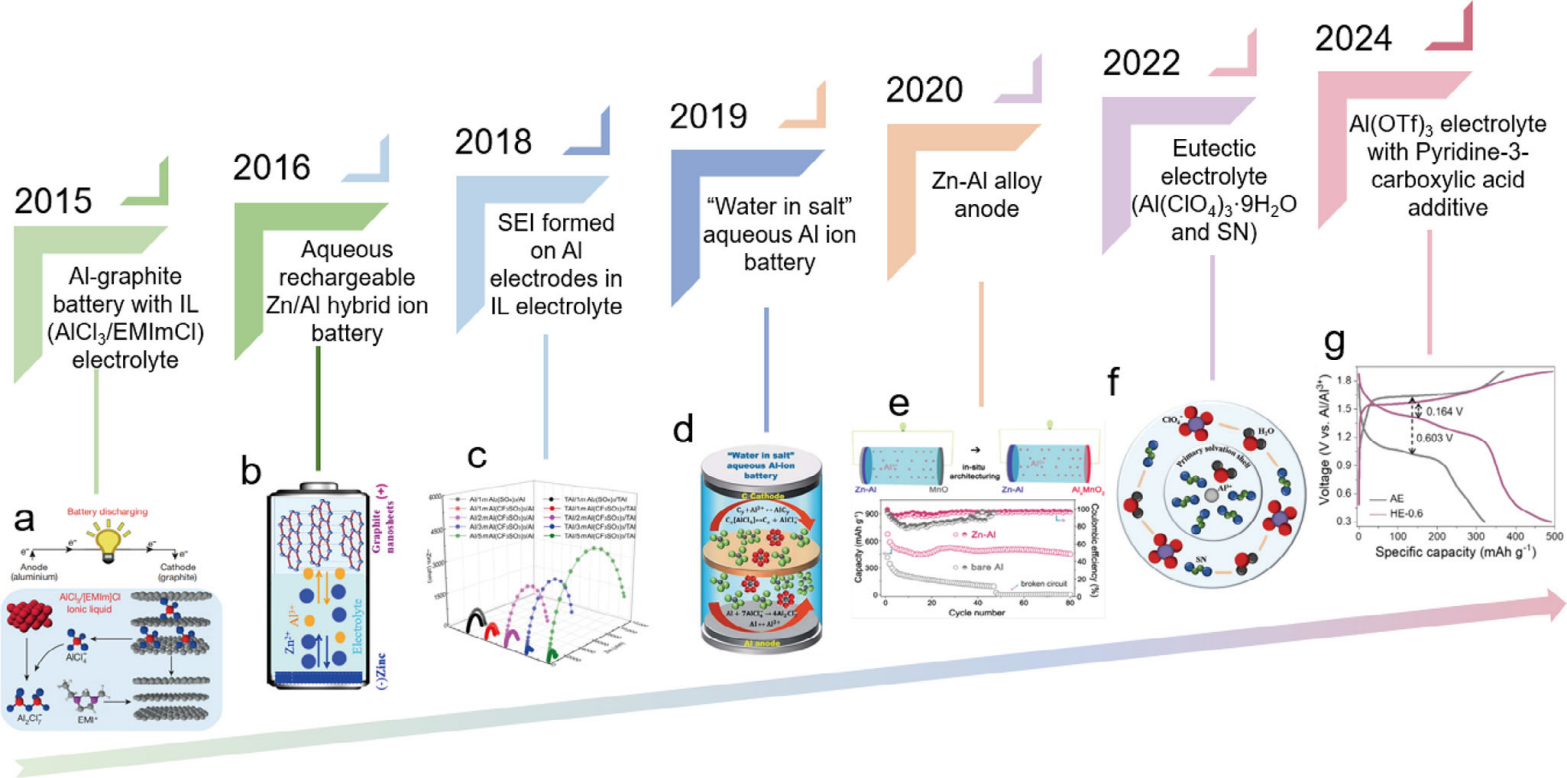
Brief development history of AAMBs. a) Al‐graphite battery with IL (AlCl3/EMImCl) electrolyte. Reproduced with permission.^[^ ^12^ ^]^ Copyright 2015, Springer Nature. b) Aqueous rechargeable Zn/Al hybrid ion battery. Reproduced with permission.^[^ ^19^ ^]^ Copyright 2016, American Chemical Society. c) SEI formed on Al electrodes in IL electrolyte. Reproduced under terms of the CC‐BY license.^[^ ^20^ ^]^ Copyright 2018, American Association for the Advancement of Science. d) “Water in salt” aqueous Al ion battery. Reproduced with permission.^[^ ^21^ ^]^ Copyright 2019, The Royal Society of Chemistry. e) Zn–Al alloy anode. Reproduced with permission.^[^ ^22^ ^]^ Copyright 2020, American Chemical Society. f) Eutectic electrolyte (Al(ClO_4_)_3_·9H_2_O and SN). Reproduced with permission.^[^ ^23^ ^]^ Copyright 2022, Wiley‐VCH. g) Al(OTf)_3_ electrolyte with Pyridine‐3‐carboxylic acid additive. Reproduced with permission.^[^ ^24^ ^]^ Copyright 2024, American Chemical Society.

To fully leverage the advantages of aluminum metal anode, aluminum metal batteries based on various electrolytes and cathode materials have been developed. As early as 2015, Dai's group used graphite as the cathode and achieved an ultra‐long cycle life (7500 cycles) and a specific capacity of ≈70 mAh g^−1^ for Al//Graphite cells using an AlCl_3_/1‐ethyl‐3‐methylimidazolium chloride ([EMIm]Cl) ionic liquid (IL) electrolyte.^[^ ^12^ ^]^ However, despite the advantages of IL‐based electrolytes over traditional organic electrolytes, such as low viscosity, high ionic conductivity, and improved safety, the high moisture sensitivity of ILs requires assembly in extremely dry environments. This significantly increases manufacturing costs, which undermines the benefits of aluminum metal anodes.^[^ ^13^ ^]^ It is worth noting that although several non‐corrosive and water‐insensitive IL‐based electrolytes have been developed in recent years, which eliminate the corrosion effect on aluminum metal, these electrolytes still require pre‐treatment of the Al foil before use to reduce the insulating effect of the oxide layer.^[^ ^14^ ^]^ Meanwhile, due to the interaction mechanism of large‐sized charge carriers (such as AlCl_4_^−^) in IL‐based electrolytes and their strong acidity, the specific capacity of graphite cathode materials is low, and the cycle lifespan of oxide cathode materials is poor.^[^ ^10^, ^13^ ^]^ From the perspective of electrolytes, the current electrolyte systems for aluminum metal batteries can be divided into non‐aqueous (mainly including IL‐based, deep eutectic solvents and molten salt electrolytes) and aqueous electrolytes.^[^ ^15^ ^]^ Compared to the high cost and stringent assembly environment requirements of non‐aqueous electrolytes, AAMBs assembled with high‐safety and low‐cost aqueous electrolytes have developed rapidly over the past decade, with a significant increase in the number of publications (Figure 1d). Although research on AAMBs is still in its early stages compared to the more extensively studied aluminum‐air batteries, the intrinsic advantages of AAMBs have already sparked increasing interest among researchers. **Figure** **2** provides a concise overview of the development history of rechargeable AAMBs, highlighting the major advancements in each period.

**Figure 3:**
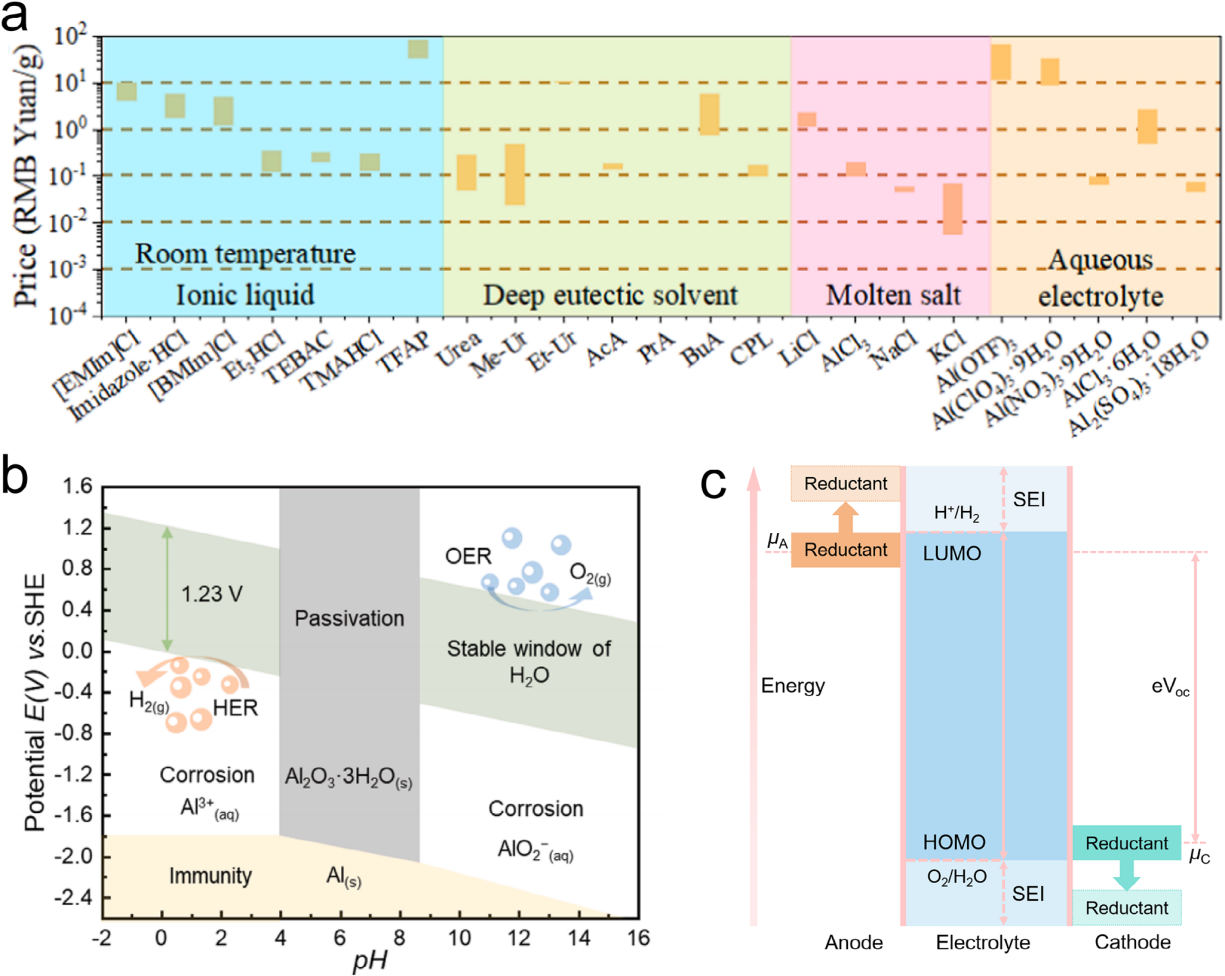
a) The cost of various electrolytes. Reproduced with permission.^[^ ^28^ ^]^ Copyright 2024, Elsevier. b) Pourbaix diagram of aluminum metal in water at 25 °C. Reproduced with permission.^[^ ^16^ ^]^ Copyright 2024, Elsevier. c) Open circuit energy diagram of aqueous electrolyte.

Benefiting from the inherent advantages of aqueous solutions, these electrolytes have intrinsic low viscosity and high ionic conductivity, granting AAMBs the potential for fast charging and high power density.^[^ ^16^ ^]^ Moreover, compared to non‐aqueous electrolytes, most aluminum salts are relatively inexpensive and do not require anoxic or dry assembly lines, which significantly reduces the manufacturing costs of both electrolytes and batteries (**Figure** **3**a). Although the current understanding of the solvation structure of aluminum ions and the energy storage mechanism in aqueous electrolytes is still in its early stage, it is closer to achieving a multivalent electron transfer reaction mechanism based on Al^3+^. However, despite the low redox potential of Al^3+^/Al and the high theoretical specific capacity endowing AAMBs with high theoretical energy density, their development and application still face some thorny issues. The current main challenge in AAMBs is the difficulty in achieving reversible Al deposition/dissolution in aqueous electrolytes. This is attributed to the low redox potential of Al^3+^/Al, which theoretically causes the hydrogen evolution reaction (HER) to occur before the electrochemical deposition of aluminum.^[^ ^17^ ^]^ In addition, Al metal can spontaneously form a dense passivation film with a large band gap when exposed to air. This passivation film not only inhibits chemical corrosion but also severely impedes the electrochemical reaction kinetics at the aluminum anode, ultimately preventing reversible aluminum plating and stripping.^[^ ^18^ ^]^ This means that aluminum metal is theoretically electrochemically inactive in aqueous electrolytes and challenging to function effectively. Therefore, to address these issues, it is necessary to explore methods to activate and stabilize the aluminum metal anode by optimizing the anode itself or the aqueous electrolyte, which is vital for achieving reversible aluminum deposition/dissolution—a critical factor in developing high‐performance AAMBs.

In recent years, AAMBs have garnered increasing attention, leading to numerous significant advancements. This progress necessitates a comprehensive and in‐depth review of the field. This review focuses on methods to activate and stabilize the aluminum metal anode in aqueous electrolytes, which are crucial for developing high‐performance AAMBs. This review begins by outlining the critical challenges faced by aluminum metal anodes and aqueous electrolytes. It then explores various strategies in two key areas: engineering the aluminum anode and optimizing the electrolyte. These strategies are essential for achieving reversible aluminum deposition and dissolution, which are vital for the battery's longevity and efficiency. Finally, the review discusses remaining challenges and future directions, providing insights into the ongoing development of AAMBs. We anticipate that this review can provide effective assistance and insights for the design and development of advanced AAMBs.

## 2 Mechanisms and Challenges for Al Metal Anodes

AAMBs have the advantages of abundant raw materials, environmental friendliness, high safety, and low manufacturing costs, making them one of the most promising candidates for new ESS. However, many key challenges have not been well addressed, primarily the difficulty in achieving stable and reversible dissolution and deposition of aluminum during battery operation.

### 2.1 Reaction Mechanism of the Al Metal Anodes

A fundamental prerequisite for achieving rechargeable AAMBs is to ensure the reversible deposition/dissolution of Al^3+^ on the aluminum metal anode. However, unlike the Zn metal anodes which are widely studied recently, the lower redox potential of aluminum leads to competition from hydrogen ions in the aqueous solution during the deposition process, making it difficult to achieve an ideal deposition process of Al^3+^.^[^ ^25^ ^]^ Furthermore, since research on AAMBs is still in its infancy, the solvation structure of the carriers and the reaction mechanisms at the anode/electrolyte interface (AEI) are not yet clear. Currently, three different theories have been proposed for the mechanisms during the deposition process of AAMBs, which are based on aluminum salts with different concentrations and types.

The mechanism of the first simple reaction involves the reversible dissolution and deposition of Al^3+^ ions on the surface of the aluminum anode. Specifically, Al^3+^ ions can also be reversibly inserted and extracted within the cathode materials. The electrochemical reaction equation on the Al metal anode is as follows:

(1) $$\mathrm{Al}↔Al^{3+}+3e^{-}$$

Recently, research on the solvation structure of aluminum ions has found that in dilute aqueous electrolytes, Al^3+^ ions typically exist in the form of hydrates. The Al^3+^ solvation structure consists of a primary solvation sheath and a secondary solvation sheath, each containing six water molecules in coordination.^[^ ^26^ ^]^ Therefore, the process of aluminum deposition first requires the de‐solvation of hydrated aluminum ions. The reversible plugging and unplugging of H^+^ replaces the plating and stripping of Al^3+^.^[^ ^27^ ^]^ The second mechanism is as follows:

(2) $${\left[\mathrm{Al}{\left(H_{2}O\right)}_{6}\right]}^{3+}↔{\left[\mathrm{Al}\left(\mathrm{OH}\right){\left(H_{2}O\right)}_{5}\right]}^{2+}+H^{+}$$

Because AlCl_3_ has a high solubility in water, it is commonly used to prepare high‐concentration electrolytes. When assembling AAMBs with such electrolytes, the carriers in the aqueous solution are in the form of AlCl_4_^−^ or Al_2_Cl_7_^−^. During deposition, the Al_2_Cl_7_^−^ anion is converted to AlCl_4_^−^. It is worth noting that during the electrodeposition process, only the Al_2_Cl_7_^−^ species can be reduced to Al, and the corresponding reaction at the anode surface is:

(3) $$4Al_{2}C{l_{7}}^{-}+3e^{-}↔7\mathrm{AlC}{l_{4}}^{-}+\mathrm{Al}$$

### 2.2 Challenges for Anodes and Aqueous Electrolytes

Due to the intrinsic advantages of aluminum metal, using aluminum metal directly in aqueous solutions as the anode can theoretically significantly reduce costs and achieve a high‐energy‐density ESS. However, aluminum metal has a low redox potential (−1.672 V vs SHE), much lower than the HER. This means that during the electroplating process, both the decomposition of the electrolyte and the hydrogen evolution will occur simultaneously at the AEI. Moreover, aluminum metal is an active material which is easily oxidized when its surface is exposed to O_2_ and water. During storage, the surface of Al metal can easily form a uniform, continuous amorphous surface layer of Al_2_O_3_ with a thickness of 2–10 nm.^[^ ^29^ ^]^ This process can be expressed as the following reaction:

(4) $$4\mathrm{Al}+3O_{2}↔2Al_{2}O_{3}$$

The formation of the oxide film inhibits the electrochemical activity of aluminum, resulting in a large contact resistance and reaction overpotential, imposing kinetic limitations on the electrodeposition/electrodissolution processes of Al.^[^ ^30^ ^]^

In addition to surface passivation, there is a continuous corrosion issue with aluminum anodes in electrochemical reactions. In AAMBs, the presence of water is a double‐edged sword. The strong coordination ability and polarity of water make it an excellent solvent that can dissolve most aluminum salts. However, the strong solubility and chemical reactivity of water lead to the dissolution and corrosion of electrode materials.^[^ ^31^ ^]^ In aqueous electrolytes, aluminum ions coordinate with active water molecules to form a hexacoordinate complex. Due to the high charge density of the Lewis acid Al^3+^, the hydrated ion radius of Al^3+^ ion is larger than that of other metal cations. Furthermore, Al^3+^ and hydroxyl groups coordinate and ionize a large amount of H^+^ in subsequent processes, resulting in various aluminum salt solutions exhibiting strong acidity and low pH.^[^ ^16^ ^]^ The specific chemical reaction equations are as follows:

(5) $$\mathrm{Al}{{\left(H_{2}O\right)}_{6}}^{3+}↔\mathrm{Al}\left(\mathrm{OH}\right){{\left(H_{2}O\right)}_{5}}^{2+}+H^{+}$$

It is generally believed that the common corrosion reaction is pitting, which is caused by anions penetrating through the passive film layer.^[^ ^32^ ^]^ The corrosion rate of aluminum in aqueous electrolytes is influenced by pH value, temperature, and conductivity.^[^ ^33^ ^]^ As shown in Figure 3b, in acidic electrolytes (pH < 4), aluminum loses electrons during discharge, forming Al^3+^ ions and generating H_2_ gas until either the aluminum or water is depleted.^[^ ^34^ ^]^ Furthermore, when corrosion occurs, dissolved oxygen in water is further exacerbated in forming a passive film due to the increased active sites and exposure of active aluminum. The continuous corrosion reaction leads to the consumption of the aluminum anode and electrolyte, as well as a decrease in battery life.^[^ ^25^ ^]^

Figure 3c shows the relative electron energy in the electrodes and electrolytes of thermodynamically stable aqueous batteries. The energy gap between the highest occupied molecular orbital (HOMO) and the lowest unoccupied molecular orbital (LUMO) defines the “stability window” of the electrolyte. Due to the influence of the passivation film and the overpotentials at the cathode and anode, the electrochemical stability window (ESW) of the aqueous electrolyte is typically higher than the decomposition voltage of pure water (1.23 V).^[^ ^35^ ^]^ Nevertheless, the narrow ESW is still not comparable to non‐aqueous electrolytes.^[^ ^36^ ^]^ This not only limits the actual output voltage and energy density of AAMBs but also largely restricts the selection of cathode materials. When the working potential of the electrode material exceeds the ESW, it will lead to the decomposition of water, producing O_2_ and H_2_ on the electrode surface. Moreover, in aqueous electrolytes, the hydrogen evolution is more competitive than the deposition of aluminum ions. This is because the standard potential of Al^3+^/Al (−1.672 V vs SHE) is much lower than that of H^+^/H_2_ (0 V vs SHE).^[^ ^37^ ^]^ The continuous occurrence of HER not only severely affects the deposition and dissolution behavior of aluminum but also leads to a decrease in coulombic efficiency (CE) and the loss of active materials. Meanwhile, in sealed batteries, the produced O_2_ and H_2_ can destroy the electrode structure, isolate the electrolyte, and even cause it to expand or even rupture. Therefore, expanding the ESW and suppressing the decomposition of water molecules on the electrode surface are the main challenges faced by aqueous electrolytes. Simultaneously, the aluminum metal anodes face severe issues such as surface passivation, corrosion, and HER, thus failing to achieve reversible deposition/dissolution behaviors.

Since the redox potential of Al^3+^/Al is −1.672 V, the deposition of aluminum metal at the anode inevitably triggers undesirable HER on the aluminum surface. Concurrently, hydrogen generation is accompanied by self‐corrosion of the anode and the formation of passivation layer, both of which diminish the utilization efficiency and lifespan of the aluminum anode and hinder the transport of Al^3+^ ions. Moreover, the continuous accumulation of hydrogen implies competition between H^+^ and Al^3+^ ions for electrons at the anode.^[^ ^38^ ^]^ This competitive HER not only leads to persistent hydrogen production and difficulties in Al^3+^ deposition but also results in a lower charge/discharge plateau. Unfortunately, the spontaneous parasitic reactions between Al metal and the electrolyte are generally more serious at high current densities or large capacities. More severe HER also implies lower aluminum deposition/stripping efficiency and shorter lifespan, which further limits the utilization of the inherent high volumetric energy density advantage of the aluminum metal anode.

**Figure 4:**
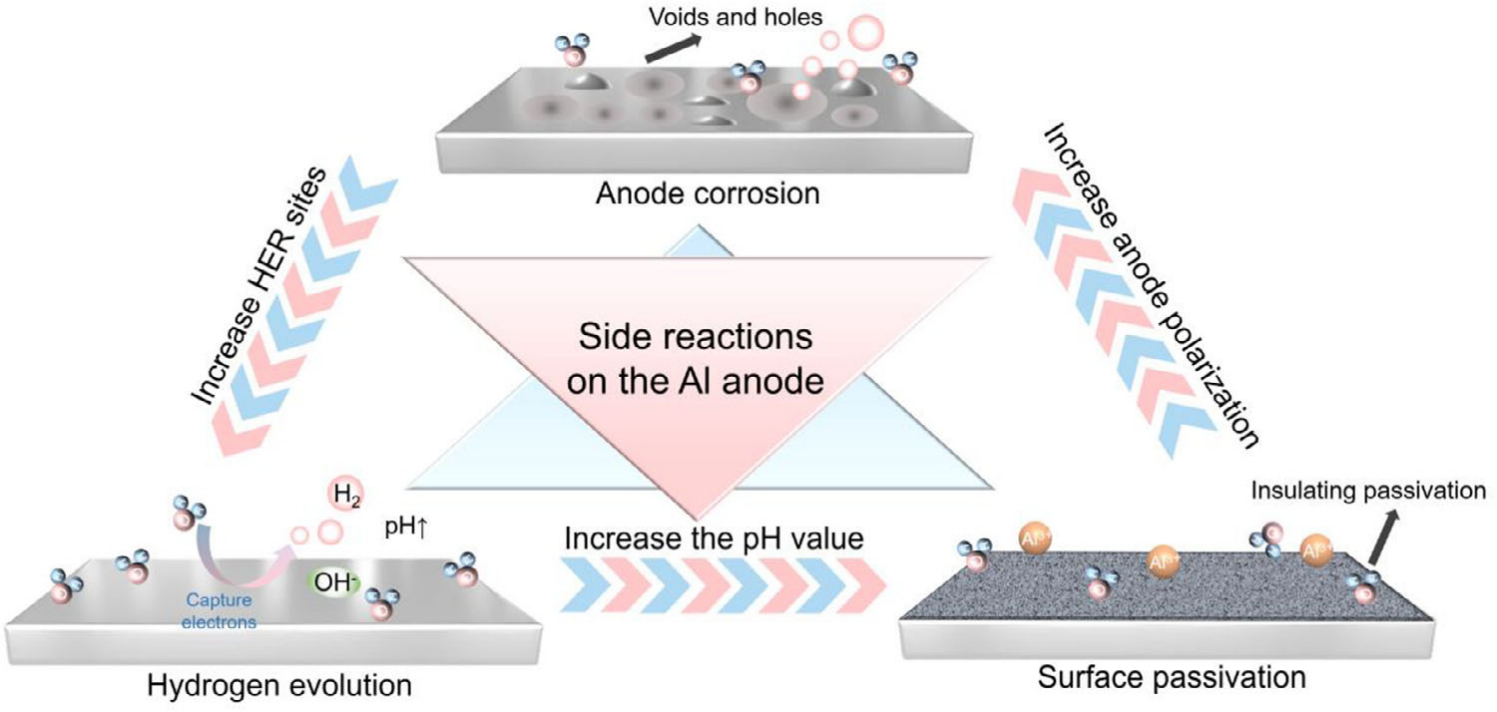
Schematic illustration of the interaction of side reactions when using aluminum anode under aqueous electrolytes.

**Figure 5:**
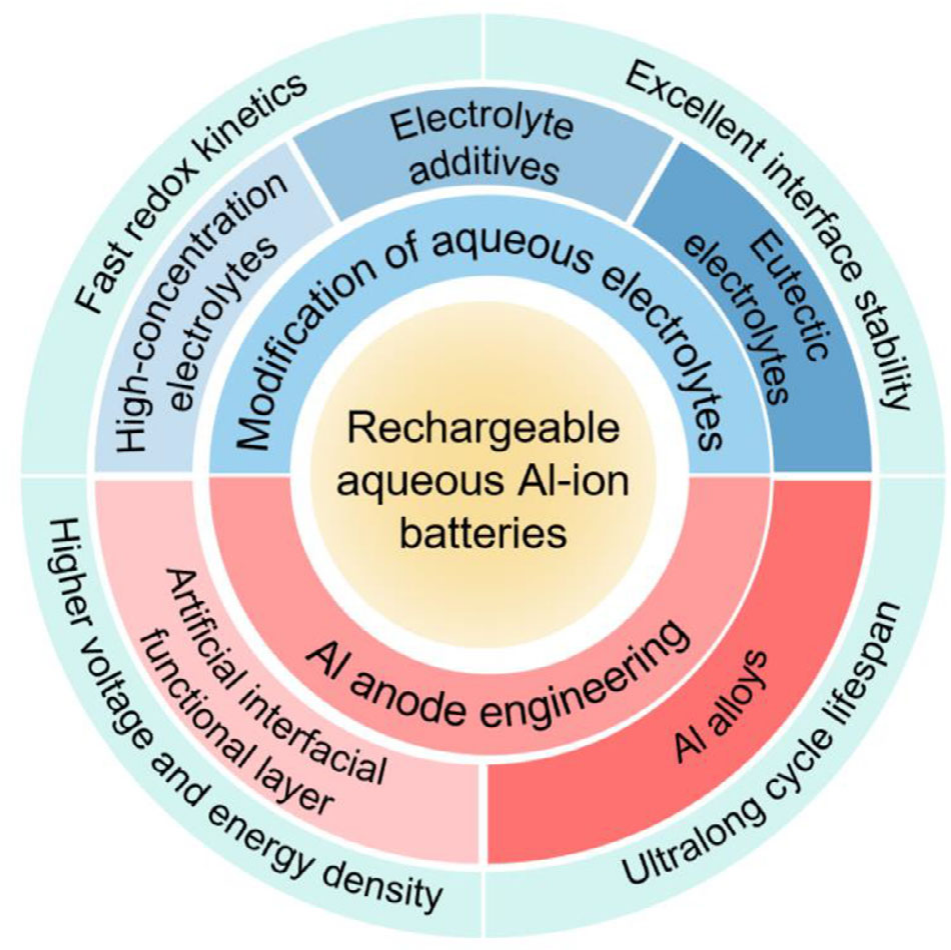
Modification strategies of activating and stabilizing Al metal anode for aqueous Al metal batteries.

It is noteworthy that when aluminum metal anodes are directly employed in AAMBs, these issues do not exist in isolation, as shown in **Figure** **4**. Parasitic HER and the non‐uniform deposition/stripping of aluminum ions cause damage and degradation of the anode surface.^[^ ^39^ ^]^ The rough electrode surface induces uneven ion flux and local current density, further exacerbating hydrogen evolution and inhomogeneous aluminum deposition. Additionally, hydrogen evolution increases the pH of the electrolyte, facilitating the passivation of the aluminum anode.^[^ ^40^ ^]^ The high voltage driving ions through the passivation layer will in turn intensify the evolution and corrosion of hydrogen gas, forming a vicious cycle. Therefore, when utilizing aluminum metal anodes, particular attention must be paid to the removal of oxides at the AEI and the uniformity of aluminum deposition, both of which significantly impact the cycling stability of the aluminum metal anode. In the past few years, researchers have invested considerable effort in the design and optimization of electrodes and electrolytes to activate and stabilize Al metal anodes (**Figure** **5**).

## 3 Al Anode Engineering

Owing to the remarkable advantages of aluminum metal, the majority of researchers are inclined to directly employ aluminum foil as the anode. However, the spontaneous formation of an oxide passivation layer on the aluminum surface in the air leads to slow rates of aluminum deposition and dissolution. Moreover, electrochemical corrosion and HER still persist. To address these challenges, it is crucial to modify the interface of aluminum or construct stable novel composite anodes. Up to now, several strategies for Al anode engineering have been proposed (**Table** **1**), mainly including the construction of artificial interfacial functional layers on the aluminum metal anode surface and the use of aluminum alloy anodes.

**Table 1:** Cyclic performance of symmetric cells using different modified anodes.

Anode	Electrolyte	Current density [mA cm^−2^]	polarization voltage [V]	Cycling life [h]	Refs.
Al treated with AlCl_3_‐[EMIm]Cl	2 m Al(OTF)_3_	0.2	0.1	50	[20]
Al treated with AlCl_3_/urea	2 m Al(OTF)_3_	0.2	0.8	40	[41]
Al treated with AlCl_3_/urea	2 m Al(OTF)_3_	0.1	≈1.5	350	[42]
Al treated with AlCl_3_‐Acm	2 m Al(OTF)_3_	0.05	0.2	300	[43]
Al coated with Mn/Ti/Zr compounds	1 m Al(OTF)_3_	0.1	≈1	300	[44]
PVDF‐Al	1 m Al(OTF)_3_	0.1	≈0.5	100	[45]
Al@α‐Al	0.5 m Al_2_(SO_4_)_3_	0.05	<0.15	800	[46]
Zn‐Al alloy	2 m Al(OTF)_3_	0.2	≈0.1	1500	[22]
Sn@Al	0.5 m Al_2_(SO_4_)_3_	0.05	<0.5	900	[47]
Al_82_Cu_18_ alloy	2 M Al(OTF)_3_	0.5	<0.05	2000	[48]
MXene/E‐Al_97_Ce_3_	2 M Al(OTF)_3_	0.5	0.054	1000	[49]

### 3.1 Artificial Interfacial Functional Layer

**Figure 6:**
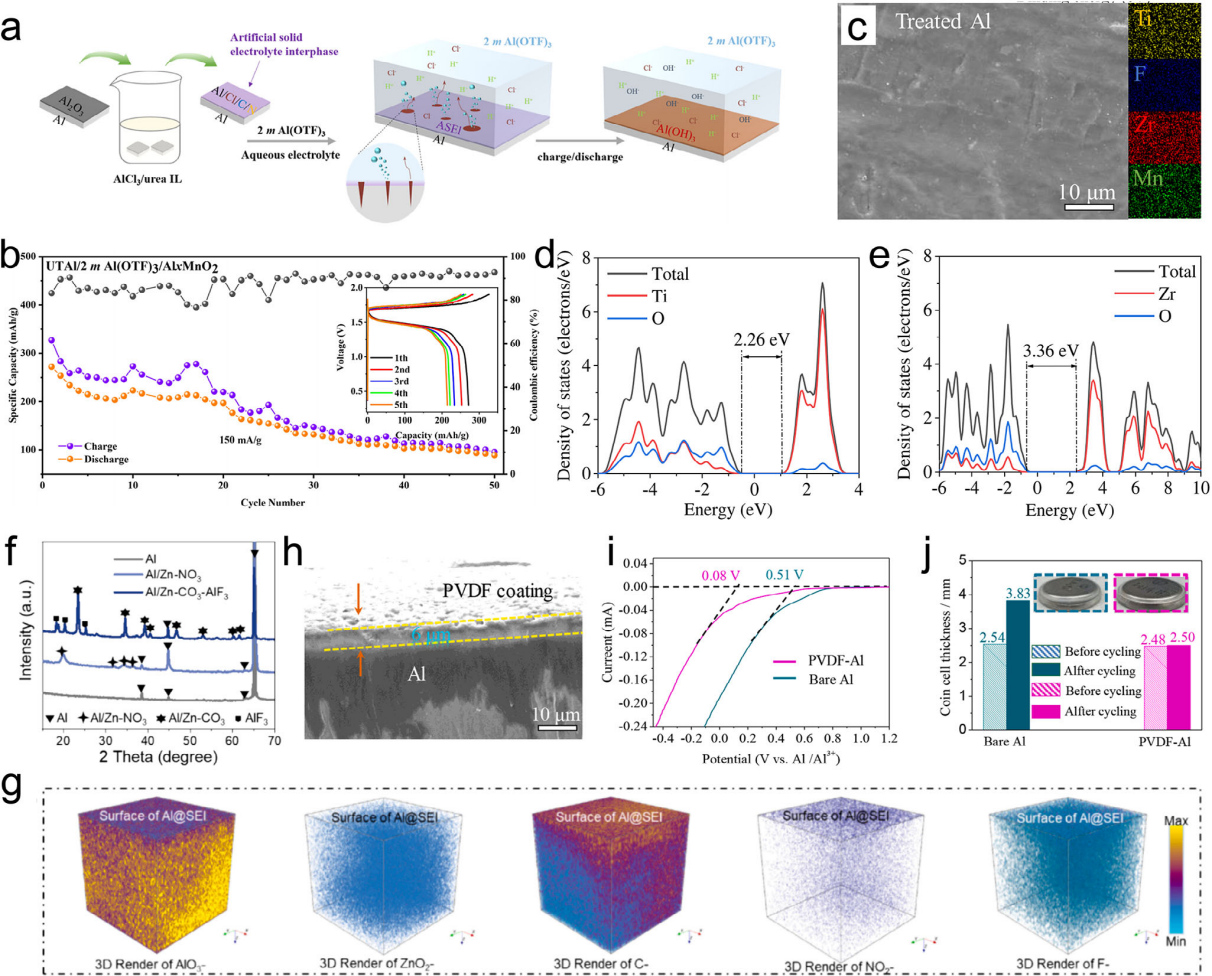
Anode engineering with artificial interfacial functional layer to improve electrochemical performance: a) Schematic diagram depicting the interphase reactions between the UTAl electrode and electrolyte. b) Long cycling performance of full cell using UTAl anode at 150 mA g^−1^. Reproduced with permission.^[^ ^41^ ^]^ Copyright 2023, American Chemical Society. c) EDX elemental mapping of treated Al metal. DOS image of d) TiO_2_ and e) ZrO_2_. Reproduced with permission.^[^ ^44^ ^]^ Copyright 2023, American Chemical Society. f) XRD patterns of bare Al, treated Al, and electrochemically activated Al electrodes. g) TOF‐SIMS analysis of treated Al anode. Reproduced with permission.^[^ ^50^ ^]^ Copyright 2024, Elsevier. h) Cross‐sectional SEM image of the PVDF coating. i) LSV curves of anode with/without PVDF layer. j) Thickness change of symmetric cells using different anodes. Reproduced with permission.^[^ ^45^ ^]^ Copyright 2022, Elsevier.

Al anode engineering is used to adjust the composition and microstructure of the aluminum metal anode surface, thereby enhancing the electrochemical stability. As is well known, maintaining the activity of the anode and achieving reversible metal deposition/dissolution in aqueous electrolytes are crucial for AAMBs. As early as 2018, Archer's group treated aluminum foil with an ionic liquid electrolyte (AlCl_3_‐[EMIm]Cl).^[^ ^20^ ^]^ The solid electrolyte interphase (SEI) derived from the ionic liquid can prevent the oxidation of Al metal and provide an acidic internal environment to maintain a fresh aluminum surface. The organic‐inorganic hybrid SEI layer effectively altered the interfacial chemistry of the Al metal anode, enabling rapid charge transfer at the AEI and realizing reversible aluminum ion plating/stripping. Since then, an increasing number of studies have focused on constructing a robust artificial interfacial functional layer on the aluminum anode, which is a key step toward achieving high‐performance AAMBs.^[^ ^32^ ^]^ In addition to ionic liquids, deep eutectic solvents can also be used to treat and remove the passivation layer on the surface of the aluminum anode, thereby maintaining the electrochemical reactivity of the Al metal anode.^[^ ^42^, ^43^ ^]^ Li et al. investigated the effects of an artificial SEI on the Al anode by immersing Al foils in an AlCl_3_/urea ionic liquid (**Figure** **6**a).^[^ ^41^ ^]^ Their results indicated that such SEI facilitated the corrosion of Al by supplying chloride anions rather than enhancing the transport of Al^3+^ ions during charge/discharge processes. The SEI substantially improved the cycling stability and electrochemical activity of cells. Based on the urea‐treated Al (UTAl) anode and Al_x_MnO_2_ cathode, the battery shows high specific capacity of 280 mAh g^−1^ and discharge operating voltage of around 1.45 V (Figure 6b). In recent years, the direct construction of SEI with excellent ion transport and corrosion resistance on the surface of aluminum metal through in situ chemical reactions has also been studied. Hao et al. employed a rapid surface passivation strategy to develop a dense passivation layer composed of Mn/Ti/Zr compounds on the Al metal anode (Figure 6c).^[^ ^44^ ^]^ The presence of electron‐insulating components, such as TiO₂ and ZrO₂, in the conversion layer effectively inhibits the corrosion of the Al electrode and the decomposition of the electrolyte (Figure 6d,e). The layered material MnO_2_ serves as a diffusion pathway for Al^3^⁺ ions, facilitating reversible aluminum deposition. Moreover, the Zr‐Ti‐based additive enhances the adhesion and stability of the conversion layer. The symmetrical cells assembled with the treated Al electrodes demonstrate stable cycling performance for over 300 cycles at a current density of 0.1 mA cm^−2^ and a capacity of 0.05 mA h cm^−2^. The in situ construction of a dense inorganic AlF_3_+Al/Zn‐CO_3_ composite with a flexible carbon shell as a SEI on the Al electrode surface was reported, which was achieved via hydrothermal and electrochemical activation in a dilute aqueous solution of Al(OTf)_3_‐Zn(NO_3_)_3_ (Figure 6f,g).^[^ ^50^ ^]^ An electrically insulating Al/Zn‐NO₃ layer initially forms on the Al anode surface through self‐polymerization reactions between Al and Zn^2+^/NO_3_^−^ ions under sealed heating conditions. Subsequent electrochemical activation triggers the decomposition of CF_3_SO_3_^−^, yielding conductive AlF_3_ and Al/Zn‐CO_3_. Simultaneously, a flexible carbon layer is generated in situ on the inner surface. The results show that the inorganic inner layer promotes Al‐ion diffusion, while the organic carbon shell prevents water permeation and ensures the stability of the SEI structure. Research has found that constructing organic polymer coatings on the surface of aluminum electrodes not only prevents water and oxygen infiltration but also guides the deposition of Al^3+^ ions on the electrode.^[^ ^51^ ^]^ Hao et al. designed a poly(vinylidene difluoride) (PVDF) coating that can effectively inhibit the permeation of free H_2_O and O_2_, thereby alleviating the corrosion issue of Al metal (Figure 6h–j).^[^ ^45^ ^]^ PVDF interacts strongly with Al^3+^ ions through the formation of F‐Al bonds, which facilitates uniform aluminum deposition. The assembled full cell (PVDF‐Al/K_2_CoFe(CN)_6_) achieves a high CE of 98.2% (at 0.1 A g^−1^ after 400 cycles).

### 3.2 Al Alloys

Apart from preparing artificial interfacial functional layers on the aluminum anode, designing aluminum alloys by selecting alloy elements with excellent corrosion resistance or conductivity can also alter the standard potential of the anode. This enhances its stability in aqueous electrolytes, allowing for the reversible deposition/dissolution of aluminum ions. Such modifications improve the electrochemical performance by reducing issues like corrosion and enhancing the efficiency of ion transport.^[^ ^52^ ^]^ Alloying elements can also promote the deposition of aluminum through an underpotential deposition process. These effects suggest that batteries using aluminum alloys may exhibit lower voltage polarization, higher discharge platforms, and improved CE. It is important to note that when inappropriate alloying elements are added to aluminum metal, the corrosion phenomena accompanied by hydrogen evolution may intensify.^[^ ^53^ ^]^ To date, elements such as zinc, tin, copper, and cerium have been preliminarily studied as typical alloying elements to enhance electrochemical activity and suppress side reactions at the AEI.

**Figure 7:**
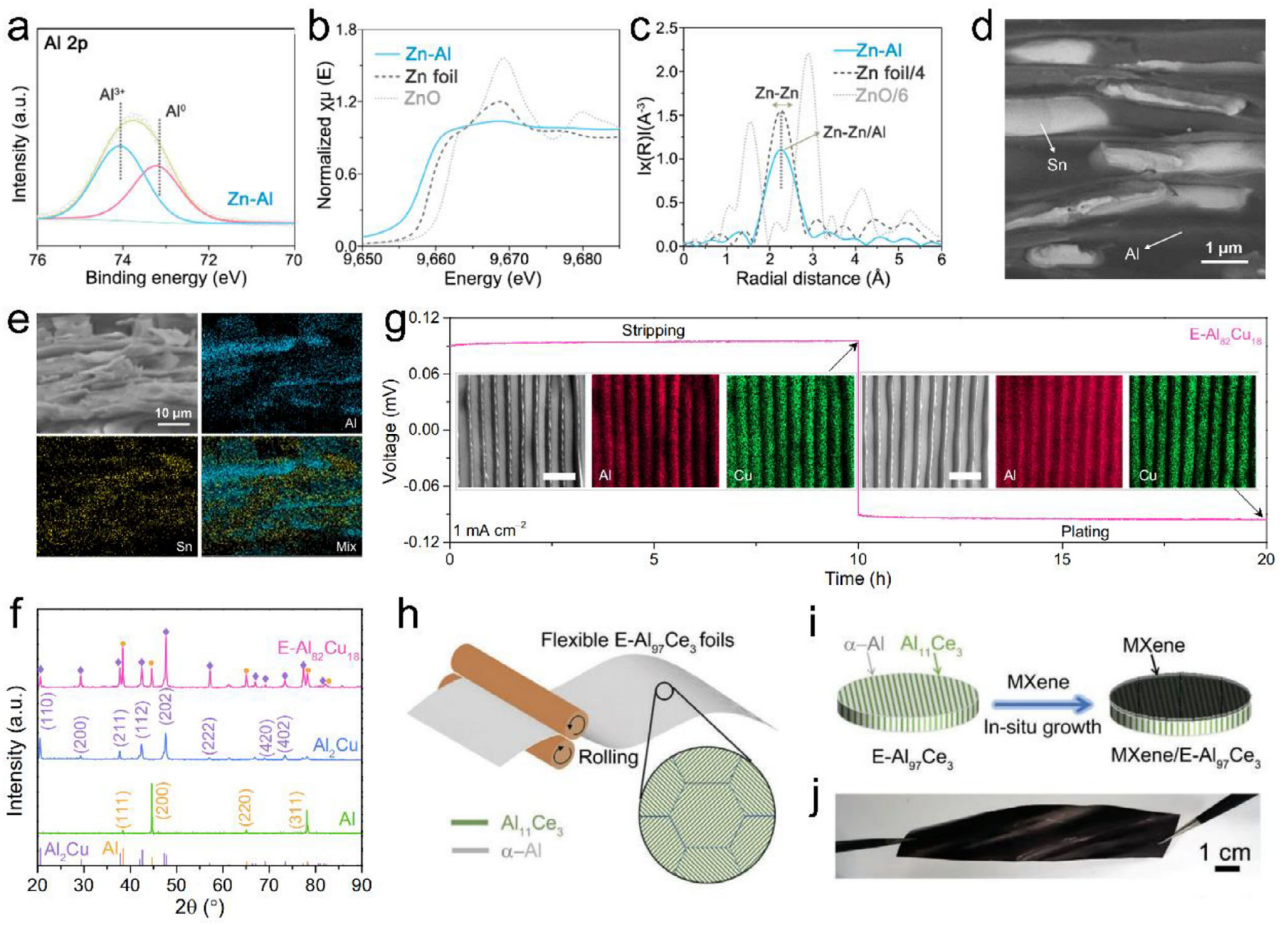
Anode engineering with designing aluminum alloys to improve electrochemical performance: a) Al 2p XPS spectrum of Zn–Al alloy anode. b) XANES spectra and c) EXAFS spectra of Zn–Al alloy and bare Zn electrodes. Reproduced with permission.^[^ ^22^ ^]^ Copyright 2020, American Chemical Society. d) Secondary electron micrographs of Sn@Al anode. e) Cross‐sectional SEM image and EDS elemental mappings of Sn@Al anode after 500 cycles. Reproduced with permission.^[^ ^47^ ^]^ Copyright 2024, Elsevier. f) XRD patterns of different electrodes. g) Typical stripping/plating voltage profile of symmetric cells using E‐Al_82_Cu_18_ electrodes. Reproduced under terms of the CC‐BY license.^[^ ^48^ ^]^ Copyright 2022, Springer Nature. Schemes for h) preparing E‐Al_97_Ce_3_ foils and i) fabrication of MXene/E‐Al_97_Ce_3_ anodes. j) Optical photographs of MXene/E‐Al_97_Ce_3_ anodes. Reproduced with permission.^[^ ^49^ ^]^ Copyright 2023, Wiley‐VCH.

In 2020, Yan et al. constructed symmetrical cells using Zn foil as the electrode and a 2 m Al(OTf)_3_ aqueous solution as the electrolyte.^[^ ^22^ ^]^ During the charging process, Al^3+^ ions in the electrolyte are deposited onto the Zn substrate and react with it to form a Zn‐Al alloy anode. X‐ray photoelectron spectroscopy (XPS) and extended X‐ray absorption fine structure (EXAFS) spectra clearly confirm the metallic states of Zn and Al, thereby verifying the formation of the Zn‐Al alloy (**Figure** **7**a–c). The addition of the alloying element Zn effectively reduces passivation and self‐discharge behavior, while enhancing the CE by suppressing the hydrogen evolution side reaction. As a result, the designed cell exhibits a high discharge voltage of 1.6 V, and an unprecedented high capacity of 460 mAh g^−1^ (after 80 cycles at 0.1 A g^−1^). Due to the stability of aluminum‐zinc alloy electrodes in aqueous electrolytes, it is commonly used as an anode that can achieve reversible deposition/dissolution of aluminum ions when researching new cathode materials.^[^ ^28^, ^54^ ^]^ Jia et al. employed a scalable folding and rolling method to fabricate Sn‐Al laminate electrodes (Sn@Al) (Figure 7d,e). In such anode, the metallic Al network functions as a reservoir for Al‐ion, ensuring a continuous supply within the electrode structure.^[^ ^47^ ^]^ Meanwhile, the Sn framework provides abundant active sites for the underpotential deposition of Al^3+^ ions, thereby enhancing the competitiveness of Al deposition over the HER. Meanwhile, the combination of Sn and Al creates localized Al/Sn galvanic couples, which effectively facilitate Al stripping. This results in reduced internal resistance and enhanced charge transfer kinetics. The batteries assembled with the Sn‐Al alloy anode demonstrate stable cycling performance over 900 h in symmetric cells and exhibit a high discharge voltage plateau of 1.5 V when paired with either Al_x_MnO_2_ cathode. Ran et al. engineered the eutectic Al_82_Cu_18_ alloy anode, featuring a lamellar nanostructure composed of alternating 𝛼‐Al and intermetallic Al_2_Cu nanolamellas (Figure 7f).^[^ ^48^ ^]^ Due to their distinct corrosion potentials, the less‐noble α‐Al lamellas function as electroactive materials to provide Al^3+^ charge carriers, while the more‐noble Al_2_Cu lamellas act as two‐dimensional nanopatterns to facilitate highly reversible Al deposition/dissolution processes at low overpotentials (Figure 7g). Consequently, such eutectic Al₈₂Cu₁₈ anode achieves remarkable cycling performance for over 2000 h at 0.5 mA cm^−2^. Additionally, a eutectic Al_97_Ce_3_ alloy uniformly coated with MXene (MXene/E‐ Al_97_Ce_3_) was developed as a reversible anode by Ran et al (Figure 7h–j).^[^ ^49^ ^]^ The E‐Al_97_Ce_3_ alloy enables reversible Al deposition/dissolution via the different corrosion potentials of the symbiotic α‐Al metal and intermetallic Al_11_Ce_3_ lamellas, while the MXene functions as a stable SEI to suppress side reactions. It is worth noting that recent reports have also raised doubts about the effectiveness of aluminum alloy anodes. Wu et al. believe that although Al–Cu alloys and Zn–Al alloys exhibit lower voltage gaps and resistance, the improvement in these properties is mainly due to corrosion reactions rather than reversible storage of aluminum ions.^[^ ^55^ ^]^ The observed gas evolution and severe corrosion phenomena in the experiment indicate that these electrodes do not have true rechargeable properties in aqueous electrolytes.

## 4 Modification of Aqueous Electrolytes

In a battery, the electrolyte serves as a crucial component, providing a pathway for ion transport and influencing the chemical reactions occurring at the electrode/electrolyte interface. Aqueous electrolytes have garnered widespread attention due to their high ionic conductivity, low viscosity, environmental friendliness, and high safety. As an integral part of AAMBs, the electrolyte not only serves as a channel for the migration of aluminum ions between the cathode and anode but also plays a crucial role in maintaining the electrochemical activity and stability of the Al metal anode. Therefore, in this section, three most prominent electrolyte modification strategies currently employed in AAMBs are summarized, including high‐concentration electrolytes, electrolyte additives, and eutectic electrolytes. The development of stable, efficient, and novel aqueous electrolytes is key to advancing the performance of AAMBs. These electrolytes help activate and stabilize the aluminum anode, enabling higher performance and longer lifespan in battery operation. The comprehensive data provided in **Table** **2** offers a detailed overview of the performance characteristics of various electrolytes and their corresponding full‐cell configurations, highlighting the impact of these modifications on the overall performance of AAMBs.

**Table 2:** Electrochemical performance of AAMBs with various electrolytes.

Electrolyte	Anode	Cathode	Current density [mA g^−1^]	Capacity [mAh g^−1^]	Cycle life (cycles)	Refs.
1 m Al(OTf)_3_	AC	CuHCF	70	69.35	3000	[56]
5 m Al(OTf)_3_	AC	FeFe(CN)_6_	150	116	100	[57]
15 m Al(ClO_4_)_3_	Al	HOPG	200	26	2000	[58]
2 m Al(OTf)_3_ + 20 m LiTFSI	Al	CuHCF	50	74.5	20	[59]
1 m Al(OTF)_3_ + 15 m LiOTF	Al	Al_x_MnO_2_⋅nH_2_O	30	160	150	[60]
0.5 m AlCl_3_ + 12 m LiTFSI	Al	K_2_V_6_O_16_⋅2.7H_2_O	4000	80.75	300	[61]
2 m Al(OTF)_3_ + 0.5 m MnSO_4_	IL treated Al	MnO_2_	100	320	65	[62]
5 m Al(OTF)_3_ + 1 m HOTF + 1 m Zn(OTF)_2_	treated Al	MnO	100	313	100	[63]
3 m Al(TFSI)_3_ + 70 mm MnSO_4_	Zn‐Al alloy	α‐MnO_2_	150	450	400	[40]
5 m Al(OTf)_3_ + 1 m H_3_PO_4_	Al	PANI	2000	51	3850	[64]
3 m Al(OTf)_3_ + 50 wt.% EMIMTfO	Zn‐Al alloy	MnO_2_‐aniline	100	250	30	[65]
1 m Al(OTf)_3_ in (DMF: DMMP: H_2_O = 6:1: 4)	Al	Al*_x_* MnO_2_	300	335	400	[66]
1 m Al(OTf)_3_ + 0.6 m PCA	Al	Al*_x_* MnO_2_	200	253	600	[24]
Al (ClO_4_)_3_: MU = 1: 4	Al	V_2_O_5_	100	320	100	[67]
Al(NO_3_)_3_·9H_2_O: Mn (NO_3_)_2_·4H_2_O: DMA = 1: 1: 20	Al	Al*_x_* MnO_2_	100	361	300	[68]
1 m Al(ClO_4_)_3_·9H_2_O in DMA + TFE	Al	CuHCF	100	86	100	[69]
Al(OTf)_3_: Gly: SG: H_2_O = 1: 8: 1: 30	Al	Prussian white	100	109	200	[70]
Al_2_(SO_4_)_3_⋅18H_2_O: urea: H_2_O = 1: 6: 10	Zn	V_2_O_5_‐G	500	165	450	[28]
Al(ClO_4_)_3_·9H_2_O: SN = 1: 12	Al	SPANI	100	185	300	[23]

### 4.1 High‐Concentration Electrolytes

Due to the different physical and chemical properties of high‐concentration electrolytes compared to traditional electrolytes, they have received significant attention.^[^ ^71^ ^]^ The salt concentration in aqueous electrolytes has a decisive impact on the solvation structure of charge carriers.^[^ ^72^ ^]^ The solvation structure further has a significant influence on the ESW, ion conductivity, and stability of AEI in the electrolyte. The electrolyte features three primary types of solvation coordination: solvent‐separated ion pairs (SSIPs), which are formed exclusively through coordination with free solvent molecules; contact ion pairs (CIPs), resulting from coordination with anions in the salt; and solvent aggregates (AGGs), arising from interactions between solvent molecules and anions.^[^ ^73^ ^]^ In traditional dilute aqueous electrolytes, SSIP is the main coordinating form.^[^ ^74^ ^]^ Besides, the low redox potential of aluminum leads to inevitable HER. As hydrogen gas is generated, an oxide passivation film will rapidly form on the surface of the active aluminum metal anode. With the increase of salt concentration in the electrolyte, the interaction between ions and ions of salt and solvent is enhanced.^[^ ^75^ ^]^ Therefore, the number of free water molecules decreases and their activity is inhibited, and the ESW of the electrolyte is broadened, which is beneficial for the reversible plating/stripping of Al^3+^ on the aluminum anode and the inhibition of HER.^[^ ^15^ ^]^ This results in high‐concentration electrolytes exhibiting significantly different electrochemical performance and anode stability compared to conventional dilute aqueous electrolytes.^[^ ^76^ ^]^

**Figure 8:**
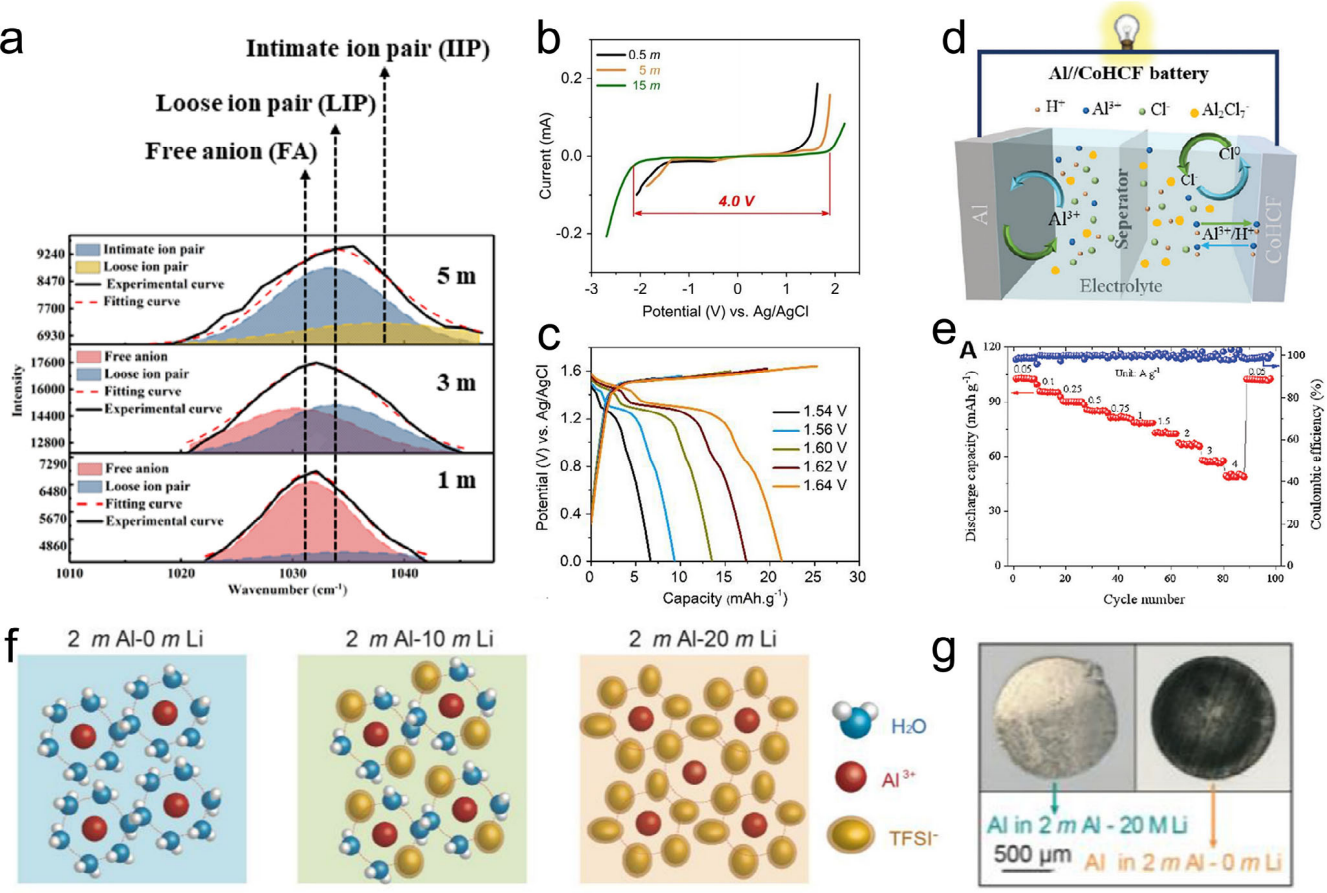
Highly concentrated aqueous electrolytes to activate and activate Al anode: a) Raman spectra of 1, 3, and 5 m Al(OTF)_3_ solution. Reproduced with permission. Copyright 2019, American Chemical Society.^[^ ^57^ ^]^ b) LSV curves of Al(ClO_4_)_3_‐based electrolytes. c) Charge/discharge curves at different cut‐off potentials of full cells. Reproduced with permission.^[^ ^58^ ^]^ Copyright 2022, Elsevier. d) Schematic illustration of working mechanism of Al//CoHCF battery. e) Capacities of Al//CoHCF batteries at different current densities. Reproduced with permission.^[^ ^77^ ^]^ Copyright 2024, Wiley‐VCH. f) Schematic illustration of LiTFSI concentration effect on solvation‐sheath structure of Al^3+^. g) Digital photos of Al anodes using different electrolytes after 360 cycles. Reproduced with permission.^[^ ^59^ ^]^ Copyright 2024, Elsevier.

Zhou et al. constructed a 5 m Al(OTF)_3_ water‐in‐salt electrolyte as a high‐performance electrolyte for AAMBs (**Figure** **8**a).^[^ ^57^ ^]^ This electrolyte possesses a broad electrochemical window of 2.65 V, which effectively suppresses the dissolution of the cathode and maintains anode activity, thereby significantly enhancing cycle stability. The full cell delivers a high discharge capacity of 116 mAh g^−1^ and remarkable cycle stability exceeding 100 cycles, with a capacity fade rate of only 0.39% per cycle. Cervenka's group reported an electrochemical investigation of a highly concentrated [Al(ClO_4_)_3_]‐based electrolyte with an extensive electrochemical stability window of 4.0 V (Figure 8b).^[^ ^58^ ^]^ The electrolyte exhibits remarkable oxidative stability exceeding 2 V and enables access to high potentials above 1.6 V versus Ag/AgCl (Figure 8c). Yuan et al. constructed a saturated aluminum chloride solution and assembled batteries using a cubic phase cobalt hexacyanoferrate (CoHCF) cathode material (Figure 8d).^[^ ^77^ ^]^ The reversible catalysis of the Cl^−^/Cl^0^ reaction at elevated potentials in a saturated AlCl₃ solution endows it with a high capacity, reaching up to 103.5 mAh g^−1^ (Figure 8e). A 2 m Al(OTf)_3_ + 20 m LiTFSI electrolyte was designed incorporating Al metal anode to address the challenges faced by AAMBs.^[^ ^59^ ^]^ Within this system, water molecules are effectively confined within the solvation structures of Li^+^ (Figure 8f). This arrangement significantly reduces the interaction between Al^3+^ and H_2_O. The unique solvation sheath structure of Al^3+^ effectively shields the Al metal anode from corrosion while suppressing the HER, thereby significantly enhancing the reversibility of the Al metal anode (Figure 8g). As a result, assembled symmetrical cells can cycle for more than 1200 h (at 200 µA/10 min).

### 4.2 Electrolyte Additives

The use of electrolyte additives is a straightforward yet highly effective method to enhance the electrochemical performance of aqueous electrolytes.^[^ ^78^ ^]^ This strategy offers significant cost advantages due to the minimal amount of additives required.^[^ ^79^ ^]^ The key role of these additives is to modify the properties of electrolytes by disrupting the hydrogen‐bonding network of water molecules or by altering the transport and diffusion kinetics of charge carriers at the interface, without compromising the aluminum metal anode's activity. This is achieved through strong interactions with water molecules or charge carriers.^[^ ^80^ ^]^ In general, the additives used in aqueous electrolytes need to meet three crucial criteria for optimal performance: (1) the presence of polar groups and lone pairs of electrons in their molecular structure to provide adsorption and hydrogen‐bonding sites; (2) excellent stability to ensure long‐term effectiveness within the electrolyte; and (3) good compatibility with both the cathode and anode materials.

**Figure 9:**
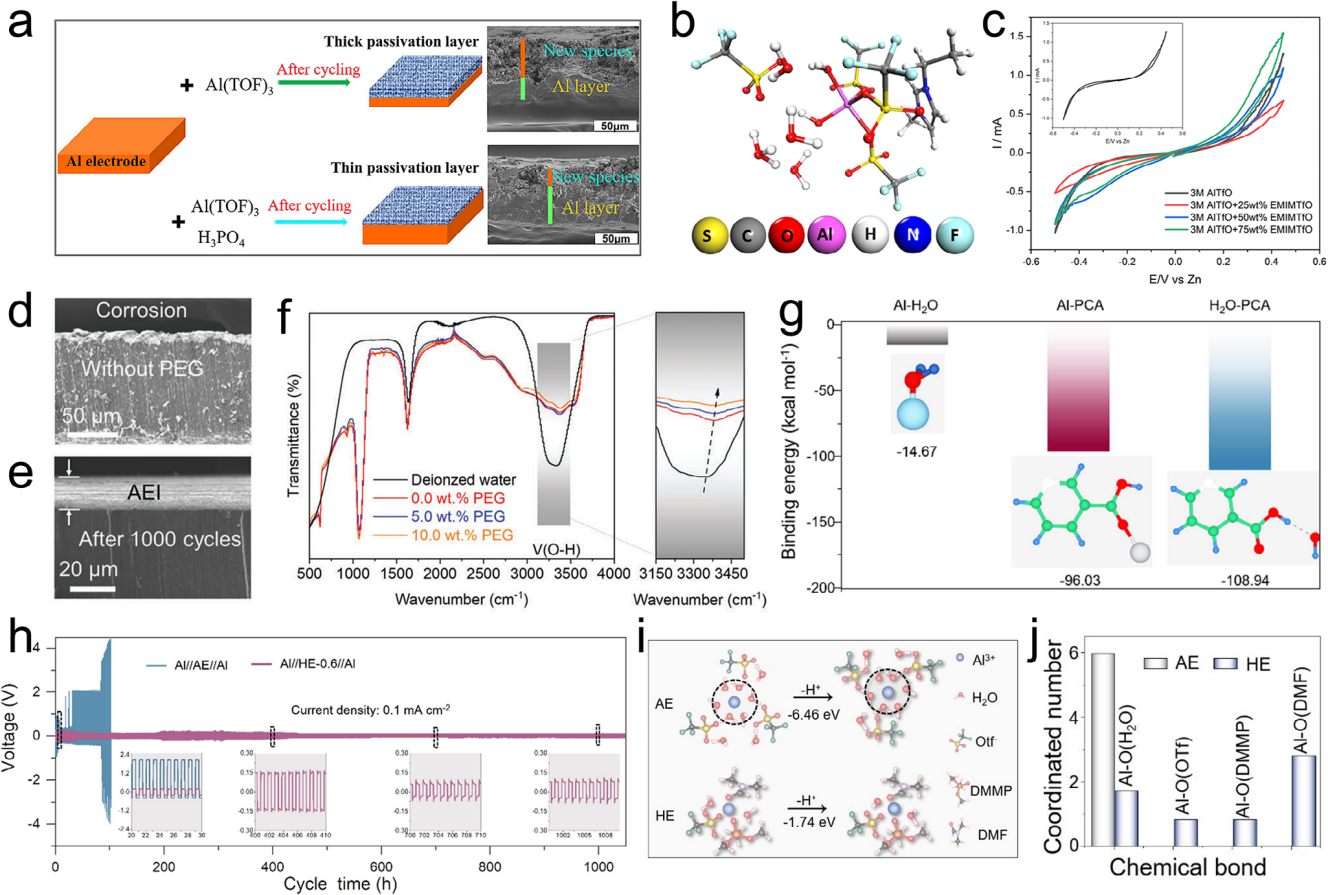
Electrolyte optimization by using electrolyte additives to activate and activate Al anode: a) Schematic diagram of passivation film generation during cycling in different electrolytes. Reproduced with permission.^[^ ^64^ ^]^ Copyright 2021, Elsevier. b) Fully relaxed geometries of Al(TfO)_3_·6H_2_O with EMIMTfO. c) CV curves of AlTfO without/with EMIMTfO on electrode. Reproduced with permission.^[^ ^65^ ^]^ Copyright 2023, American Chemical Society. The SEM images of Al anodes after cycling in electrolyte d) without and e) with PEG. f) The FTIR spectrum of different electrolytes. Reproduced with permission.^[^ ^81^ ^]^ Copyright 2023, Wiley‐VCH. g) Comparison of the absorption energies of different molecules on the Al(111) crystal plane. h) Long‐term performance of symmetric cells at 0.1 mA cm^−2^. Reproduced with permission.^[^ ^24^ ^]^ Copyright 2024, American Chemical Society. i) Deprotonation energy from solvation sheath of different electrolytes calculated from density functional theory. j) Coordination number of aluminum ions with different species in different electrolytes. Reproduced with permission.^[^ ^66^ ^]^ Copyright 2024, Wiley‐VCH.

Zhi's group identified that the formation of high‐impedance passivation layers on the aluminum anode surface during cycling is a key factor contributing to the performance degradation of AAMBs.^[^ ^64^ ^]^ To address this issue, they developed a hybrid electrolyte composed of Al(OTF)_3_ and H_3_PO_4_. This hybrid electrolyte not only mitigates the formation of passivation layers on the Al surface but also effectively reduces the strong charge density of Al^3+^ ions, thereby facilitating rapid reaction kinetics (**Figure** **9**a). As a result, the assembled Al//PANI full cell demonstrated a capacity retention rate of 58% after 3850 cycles. Ionic liquids can not only be used to treat oxides on the surface of aluminum metal and generate multifunctional SEI but also serve as electrolyte additives to alter the Al solvation dynamics. The incorporation of 1‐ethyl‐3‐methylimidazolium trifluoromethylsulfonate (EMIMTfO) was found to modify the aluminum solvation structure in the aqueous Al(TfO)_3_ electrolyte by reducing the coordination number of the solvation shells.^[^ ^65^ ^]^ This alteration significantly impacted and enhanced the aluminum deposition and stripping processes on the anode. Specifically, water molecules can coordinate with the central Al^3+^ ion, and their deprotonation may lead to the formation of AlOH species; the presence of EMIM^+^ and TfO^−^ ions can promote the formation of H_3_O^+^ species and alter the solvation environment around the Al^3^⁺ ions (Figure 9b). Consequently, these changes in the Al solvation environment distinctly influenced the deposition and stripping of Al, as illustrated in Figure 9c. Tao et al. designed a semi‐hydrogel electrolyte (PEG‐Al@H), which comprises polyethylene glycol (PEG) as the organic polymer backbone, aluminum perchlorate as the electrolyte component, and perchloric acid as an auxiliary regulator.^[^ ^81^ ^]^ During the charging process, an SEI is generated through the polymerization of PEG during the de‐solvation of Al ions (Figure 9d,e). It effectively suppresses side reactions associated with rapid kinetics and shields the Al metal anode from severe corrosion. Additionally, PEG disrupts the hydrogen bonding network of the solvent molecules in the electrolyte, thereby extending the operational temperature range of AAMBs (Figure 9f). Full cell matched with potassium manganese hexacyanoferrate (KMF) demonstrates a specific capacity of 90 mAh g^−1^ after 20000 cycles with a high CE of over 95%. Zhao et al. demonstrated that the water activity in the electrolyte can be effectively reduced by optimizing the Al^3+^ solvation structure through the intercalation of polar pyridine‐3‐carboxylic acid (PCA) in an aluminum trifluoromethanesulfonate aqueous solution.^[^ ^24^ ^]^ PCA molecules have high polarity and electron‐rich regions, specifically the N═O and C═O groups. These functional groups can preferentially interact with Al ions, effectively displacing water molecules from the Al^3+^ solvation shell. Additionally, PCA exhibits strong interactions with water. By limiting the activity of water molecules, the generation of hydrogen gas from H_2_O decomposition, as well as other undesired side reactions, is significantly suppressed. Moreover, the Al electrode surface exhibits a higher affinity for PCA compared to H_2_O (Figure 9g). This preferential adsorption increases the nucleation overpotential and facilitates symmetrical Al stripping and deposition. A specific concentration of PCA (0.6 m, where m denotes molality (mol kg^−1^)) in 1 m Al(OTf)_3_ electrolyte significantly enhances the cyclic stability of Al plating/stripping (1000 h at 0.1 mA cm^−2^) (Figure 9h). More recently, Zhao et al. proposed enhancing the electron density of H_2_O protons to intensify the electrostatic interactions between hydrogen bonds in H_2_O, thereby reducing the reactivity of water in AAMBs.^[^ ^66^ ^]^ Dimethylformamide (DMF) is selected as an additive due to its high polarity, excellent compatibility, and high electrochemical stability, characterized by an electron‐rich C═O group. Additionally, the oxygen and nitrogen atoms in DMF possess lone pairs of electrons that can coordinate with Al^3+^ ions to form stable complexes (Figure 9i,j). By limiting the activity of water molecules, such composite electrolytes can suppress undesired HER and other side reactions resulting from water decomposition. Furthermore, dimethyl methylphosphonate (DMMP) was added to this electrolyte system, which not only significantly reduces the flammability of the mixed electrolyte but also leverages its electron‐rich P = O group to interact with solvated water molecules. The Al//Al_x_MnO_2_ full cell using the hybrid electrolyte showed high capacity of 335 mAh g^−1^ after 400 cycles.

### 4.3 Eutectic Electrolytes

Eutectic electrolytes are formed by the combination of two or more components that, through specific intermolecular interactions—such as hydrogen bonding, Lewis acid‐base interactions, and van der Waals forces—create a cohesive structure. These interactions become stronger than the intrinsic forces within the individual components, leading to a unified system^[^ ^82^ ^]^ Among these, hydrated eutectic electrolytes (HEE) have attracted significant attention in recent research due to their high feasibility and tunability in preparation.^[^ ^83^ ^]^ Compared to organic and ionic liquid electrolytes, eutectic electrolytes offer significant advantages in terms of environmental compatibility and cost‐effectiveness.^[^ ^84^ ^]^ Eutectic electrolytes typically exhibit high ionic conductivity and low water content, which enhances the ESW, stabilizes the AEI, and improves cycling stability. It is worth noting that some eutectic electrolytes can also generate multifunctional SEI on the anode surface during the cycling process, further regulating aluminum ion kinetics and maintaining reaction activity of Al anodes.^[^ ^85^ ^]^ Different from solvent regulation strategies, hydrophilic eutectic electrolytes focus on blending aluminum salts with water or other hydrogen bond donors (e.g., polyols, amide, carboxylic acids, and sulfone) at specific ratios to form low‐melting eutectic mixtures, thereby optimizing electrolyte performance.^[^ ^23^ ^]^

**Figure 10:**
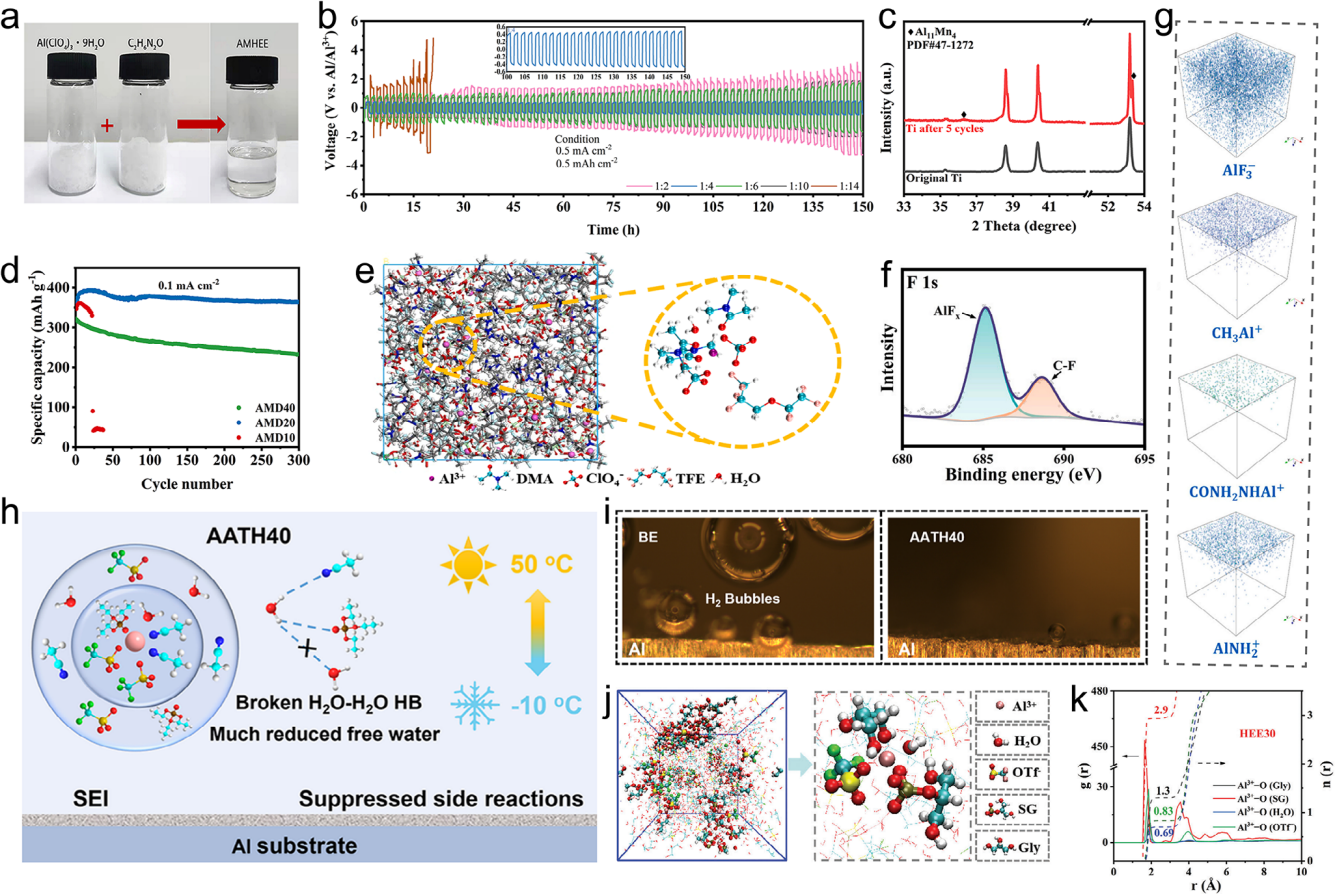
Electrolyte optimization by designing eutectic electrolytes to activate and activate Al anode: a) Preparation procedure of the AMHEE. b) Galvanostatic cycling performance of symmetric cell using AMHEE at 0.5 mA cm^−2^. Reproduced under terms of the CC‐BY license.^[^ ^67^ ^]^ Copyright 2023, Springer Nature. c) Cycling performance of cathode‐free AAMBs at 0.1 mA cm^−2^. d) XRD patterns of bare and cycled Ti foils using HEE. Reproduced with permission.^[^ ^68^ ^]^ Copyright 2024, Elsevier. e) 3D and partially enlarged snapshots of ADT45. f) F 1s XPS spectra of Al anode after cycling in ADT45. g) 3D images of depth profile for main species by TOF‐SIMS test. Reproduced with permission.^[^ ^69^ ^]^ Copyright 2024, Elsevier. h) Schematic diagram of electrolyte mechanism (AATH40) in a wide temperature range. i) In situ observation of HER at the AEI in different electrolytes. Reproduced with permission.^[^ ^87^ ^]^ Copyright 2024, American Chemical Society. j) 3D snapshot obtained from the MD simulations and the solvation structure of Al^3+^ of HEE30 electrolyte. k) RDFs and coordination number of Al^3+^ in HEE30 electrolyte. Reproduced with permission.^[^ ^70^ ^]^ Copyright 2024, Wiley‐VCH.

Meng et al. proposed a chloride‐free aluminum‐based HEE for rechargeable AAMBs, composed of aluminum perchlorate nonahydrate and neutral methylurea (MU) ligands.^[^ ^67^ ^]^ The coordination between Al^3+^ and MU induces a deep eutectic effect, transforming the two solid components into a liquid HEE (**Figure** **10**a). The resultant Al(ClO_4_)_3_·9H_2_O/MU hydrated deep eutectic electrolyte (AMHEE) is cost‐effective, non‐corrosive, environmentally benign, and highly air‐stable. In this AMHEE, water and MU molecules coordinate with Al^3+^ ions. With an optimized molar ratio of aluminum perchlorate to MU (1:4), a unique solvation structure of [Al(MU)_2_(H_2_O)_4_]^3+^ is formed, enabling stable and reversible aluminum plating/stripping. As a result, the aluminum electrode demonstrates excellent cycling stability, maintaining performance for over 150 h at a current density of 0.5 mA cm^−2^ (Figure 10b). N, N‐dimethylacetamide (DMA) with high polarity and high dielectric constant is also commonly used as a component of HEE. A cost‐effective and air‐stable ternary hydrated HEE for cathode‐free AAMBs was reported, which is composed of Al(NO_3_)_3_·9H_2_O, Mn(NO_3_)_2_·4H_2_O, and DMA.^[^ ^68^ ^]^ An optimized ratio of Al(NO_3_)_3_·9H_2_O and Mn(NO_3_)_2_·4H_2_O to DMA (1:1:20) effectively suppresses water activity and forms a unique solvation structure, where both water and DMA molecules coordinate with metal cations. This structure enhances reaction kinetics and improves the cycling stability of AAMBs. Moreover, the presence of both Al^3+^ and Mn^2+^ in the HEE enables efficient plating/stripping of Al‐Mn alloy on the anode (Figure 10c). Assembled cathode‐free full cell delivers outstanding electrochemical performance with a high discharge capacity of 361 mAh g^−1^ (Figure 10d). Adding components that can decompose and produce SEI to HEE can further improve the stability of aluminum metal anode. Fu's group further designed a HEE (named ADT45) for AAMBs, which is formulated from hydrated aluminum perchlorate, DMA, and 1,1,2,2‐tetrafluoroethyl‐2,2,3,3‐tetrafluoropropyl (TFE) diluent.^[^ ^69^ ^]^ Comprehensive spectroscopic analyses and theoretical calculations suggest that TFE creates a localized high‐concentration environment within the electrolyte (Figure 10e). This environment not only enhances ionic conductivity but also mitigates side reactions by forming an AlF_3_‐rich SEI (Figure 10f,g). By further optimizing the solvation structure of eutectic electrolytes or constructing a high concentration local environment, eutectic electrolytes can possess unique characteristics which can meet wide temperature range operation.^[^ ^86^ ^]^ Luo et al. constructed a HEE (AATH40), formulated from Al(OTf)₃, acetonitrile (AN), triethyl phosphate (TEP), and H_2_O, to enhance the electrochemical performance of AAMBs across a wide temperature range (Figure 10h). Integrating molecular dynamics simulations with spectroscopic analysis, they elucidated that AATH40 adopts a less‐water‐solvated structure [Al(AN)_2_(TEP)(OTf)_2_(H_2_O)]^3+^. This unique structure effectively suppresses side reactions, lowers the freezing point, and broadens the ESW of the electrolyte. Moreover, a protective SEI is generated through the reductive decomposition of TEP and OTf^−^, which effectively suppresses both corrosion and HER (Figure 10i). As a result, AAMBs employing this electrolyte demonstrate significantly enhanced cycling stability over a temperature range of ‐10 to 50 °C. Recently, Zhang et al. engineered an HEE using Al(OTf)₃, glycerol (Gly), sodium beta‐glycerophosphate pentahydrate (SG), and H_2_O to enhance the stability of aluminum anodes across a broad temperature range of −20 to 60 °C.^[^ ^70^ ^]^ The incorporation of SG and Gly creates a distinctive solvation structure that lowers the freezing point of the electrolyte, widens the ESW, and mitigates HER (Figure 10j,k). Additionally, Gly and SG promote the formation of a SEI layer composed of both organic and inorganic components on the aluminum surface, effectively curbing parasitic side reactions. Symmetric Al/Al cells demonstrate outstanding cycling stability for 1000 h at 0.05 mA cm^−2^ at 25 °C, and extended cycling lifespan exceeding 500 and 1000 h at −20 and 60 °C, respectively.

## 5 Optimization Strategies of Activating and Stabilizing Al Metal Anode

**Figure 11:**
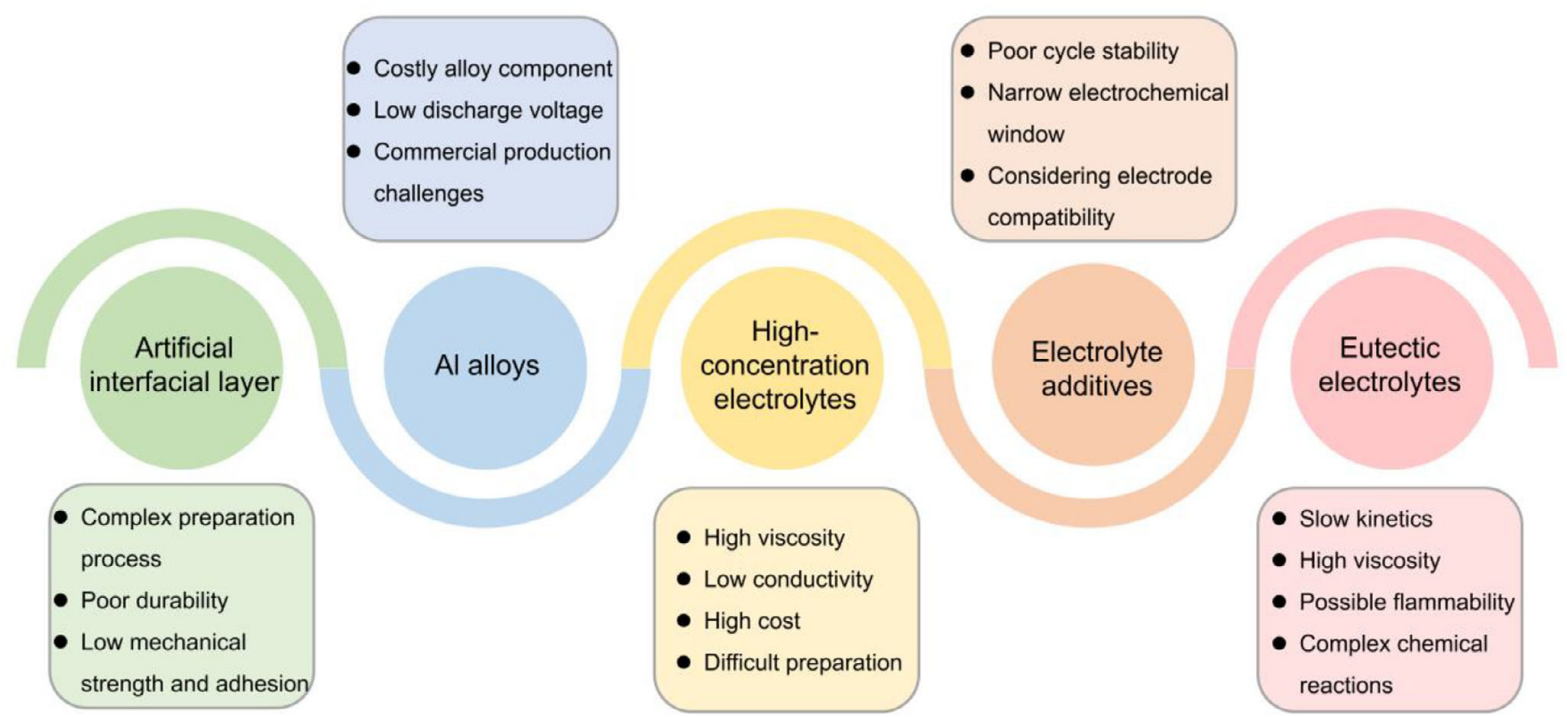
Challenges of various strategies in AAMBs.

The above discussion emphasizes the effectiveness of various optimization strategies for aluminum anode engineering and electrolyte modification. However, the current strategies for aluminum anode engineering or electrolyte modification still face several challenges, which are summarized in **Figure** **11**. In response to these challenges, feasible design directions and considerations are proposed.

### 5.1 Artificial Interfacial Functional Layer

Forming a protective layer on the surface of aluminum metal using chemical or physical methods is a promising approach to remove the passivation oxide layer and prevent further oxidation, which can improve the performance of aluminum‐based electrochemical systems. However, several challenges remain regarding the stability and mechanism of action of these artificial interfacial functional layers during aluminum deposition and dissolution. Moreover, the stability under acidic environments or long‐term cycling conditions also needs further exploration. For example, high acidity may degrade the interface layer over time, leading to the reformation of the oxide passivation layer and a reduction in performance. During multiple charge and discharge cycles, it is challenging to maintain the integrity of the artificial interface layer. The repeated stripping and plating of aluminum ions can cause mechanical stress, leading to the cracking or degradation of the interface layer. Thus, it is important to further optimize the performance, explore the mechanism of action of the interface layer, and find intuitive evidence of aluminum metal deposition. In addition, the artificial interface layer must be compatible with the electrolyte used in the battery system. Incompatibility could lead to side reactions between the interface layer and the electrolyte, reducing its effectiveness. Achieving a uniform and consistent thickness of the artificial interface layer is crucial for optimal performance. Variations in thickness may result in uneven protection and inconsistent electrochemical behavior. While constructing a stable and reliable interface layer, it is also essential to consider the material and process costs, which are vital for the future commercial feasibility of AAMBs.

### 5.2 Al Alloys

Aluminum alloys are gaining significant attention as promising anode materials for AAMBs due to their electrochemical compatibility between the alloying elements. These alloys exhibit favorable reaction kinetics and superior cycling stability, making them ideal candidates for improving the overall performance of AAMBs. One of the major advantages of using aluminum alloy anodes is the reduction in voltage hysteresis observed in galvanostatic charge/discharge tests. This results in extended cycle lifespan of the batteries, making them more durable over long periods of usage. However, despite these benefits, the use of alloy anodes introduces some trade‐offs. Discharge potential of the full cell is often sacrificed when alloy anodes are employed, leading to a reduction in energy density. This is a critical concern, as energy density directly affects the overall capacity and efficiency of the battery. To address this issue, careful selection of alloying elements is crucial, as different alloys can exhibit varying impacts on the overall performance of the anode. Another area of concern is the unclear mechanisms of the redox processes involving Al^3+^ and H^+^ ions on the alloy anodes. The exact nature of these processes—whether they result in reversible deposition and dissolution of Al^3+^ ions—is still under debate in the scientific community. Understanding these mechanisms is crucial for optimizing the alloy's behavior during charge/discharge cycles and improving its long‐term stability. Therefore, the types and kinds of alloying elements should be carefully selected, and the AEI should be thoroughly explored to determine the actual reactions occurring at the interface, thereby ensuring the functional stability of alloy anodes. During the selection of alloying elements, it is also necessary to balance the anode's corrosion resistance and reaction kinetics. Some metals with strong inertness in aqueous solutions may lead to a decrease in the overall conductivity, volumetric energy density, and chemical reaction kinetics of the electrode, which is detrimental to the inherent advantages of the aluminum metal anode. To enhance the feasibility of aluminum alloy anodes in practical applications, the cost of raw materials, preparation processes, and processing must also be considered. Although some precious metals exhibit high chemical stability and conductivity, their use in the large‐scale preparation of alloyed aluminum anodes still requires consideration of reducing their usage or finding alternatives to control costs.

### 5.3 High‐Concentration Electrolytes

In high‐concentration electrolytes, insufficient number of H_2_O molecules is affected by hydration effect. Moreover, the solvation structure is dominated by anions, which replace the coordinated water molecules due to the high concentration of both anions and cations. This leads to a reduction in free water molecules on the Al anode surface, forming a stable electrochemical interface that prevents corrosion and passivation caused by water molecule decomposition. This has a positive effect on maintaining the activity of the aluminum metal electrode and constructing a multifunctional SEI on the surface. However, while high‐concentration electrolytes offer advantages, they also come with several challenges. One of the main issues is the increased viscosity of the electrolyte due to the high concentration of salts. This higher viscosity can reduce the wettability of the electrolyte on electrode materials, which may negatively impact ion diffusion efficiency and overall battery performance. The increase in viscosity may hinder the effective movement of ions within the electrolyte, leading to slower charge/discharge cycles and reduced efficiency. Another challenge is the cost and toxicity associated with the preparation of high‐concentration electrolytes. The process requires large quantities of metal salts, some of which (especially organic aluminum salts) can be expensive and highly toxic. The need for ultrasonication and heating to facilitate the dissolution of these salts adds complexity to the preparation process, making it more time‐consuming and difficult to scale up for industrial applications.

### 5.4 Electrolyte Additives

The key to achieving reversible electrochemical electroplating/stripping of aluminum in aqueous electrolytes is to change the deposition potential of aluminum and suppress the reactivity of aqueous electrolytes. Although some acidic and organic additives can activate the dissolution of aluminum metal, the in‐depth reaction mechanism of SEI on Al^3+^ deposition is still unclear and needs further exploration. Meanwhile, due to the strong passivation tendency of aluminum metal in aqueous solutions, the effectiveness of additives is still limited during long‐term cycling. Over time, the additives may not provide sufficient benefits to counteract the formation of this oxide layer, limiting the potential for consistent, reversible electroplating/stripping. In addition, due to the complex issues at the electrode/electrolyte interface, it is crucial to ensure the compatibility between the electrolyte and electrode materials, especially when selecting additives. The synergistic effect between modifications to the anode and the electrolyte needs to be carefully considered to ensure optimal performance. This requires a balanced approach where both the anode and electrolyte are engineered to work together to enhance the stability and efficiency of the electrolyte system. Finally, due to the low dosage of additives, the ESW of electrolytes cannot be significantly expanded, and the inherent advantages of aluminum metal cannot be fully utilized. Additives or cosolvents that strongly interact with water can be explored to suppress the reactivity of water and expand the ESW. The development of multifunctional electrolytes is also a challenge for future AAMBs.

### 5.5 Eutectic Electrolytes

Hydrated eutectic electrolytes offer significant advantages over traditional organic electrolyte systems, particularly in terms of enhanced safety and potential for better performance in certain applications. However, there are still challenges to address regarding their ion conductivity and viscosity, which are critical factors for efficient electrochemical processes. The eutectic electrolytes formed by the interaction between organic aluminum salts and Lewis acid‐base typically contain larger ions and relatively free volumes, leading to lower conductivity and higher viscosity, especially at room temperature. These properties may hinder the overall efficiency of the electrolyte in practical applications. Furthermore, the electrochemical processes occurring in aqueous systems are significantly more complex than those in organic electrolyte systems.^[^ ^88^ ^]^ When eutectic electrolytes are composed of multiple components, their complex composition can alter the types and activities of redox reactions occurring within the system. This adds complexity to the optimization of these electrolytes for specific applications. The flammability and toxicity of these electrolytes are also important considerations during their preparation and usage, as they must be safe for long‐term operation and in various environmental conditions. For the successful development of advanced eutectic electrolytes, a rational design approach is crucial. This should take into account the Al^3+^ solvation structures, ion transfer mechanisms, and the electrochemical reaction pathways that are involved in these systems. To ensure good performance, the ligand should possess appropriate polarity to form strong interactions with the chosen aluminum salt, thereby lowering the melting point of the system and forming a liquid electrolyte. There should be strong intermolecular interactions between the ligand and metal salt, such as hydrogen bonds, Lewis acid‐base interactions, or van der Waals forces, to enhance the stability and ionic conductivity of the electrolyte. Additionally, it is essential that the formed eutectic electrolyte possesses good electrochemical stability to meet the demand for a high ESW, which is critical for maximizing the voltage range in energy storage systems.

## 6 Conclusion and Future Prospects

**Figure 12:**
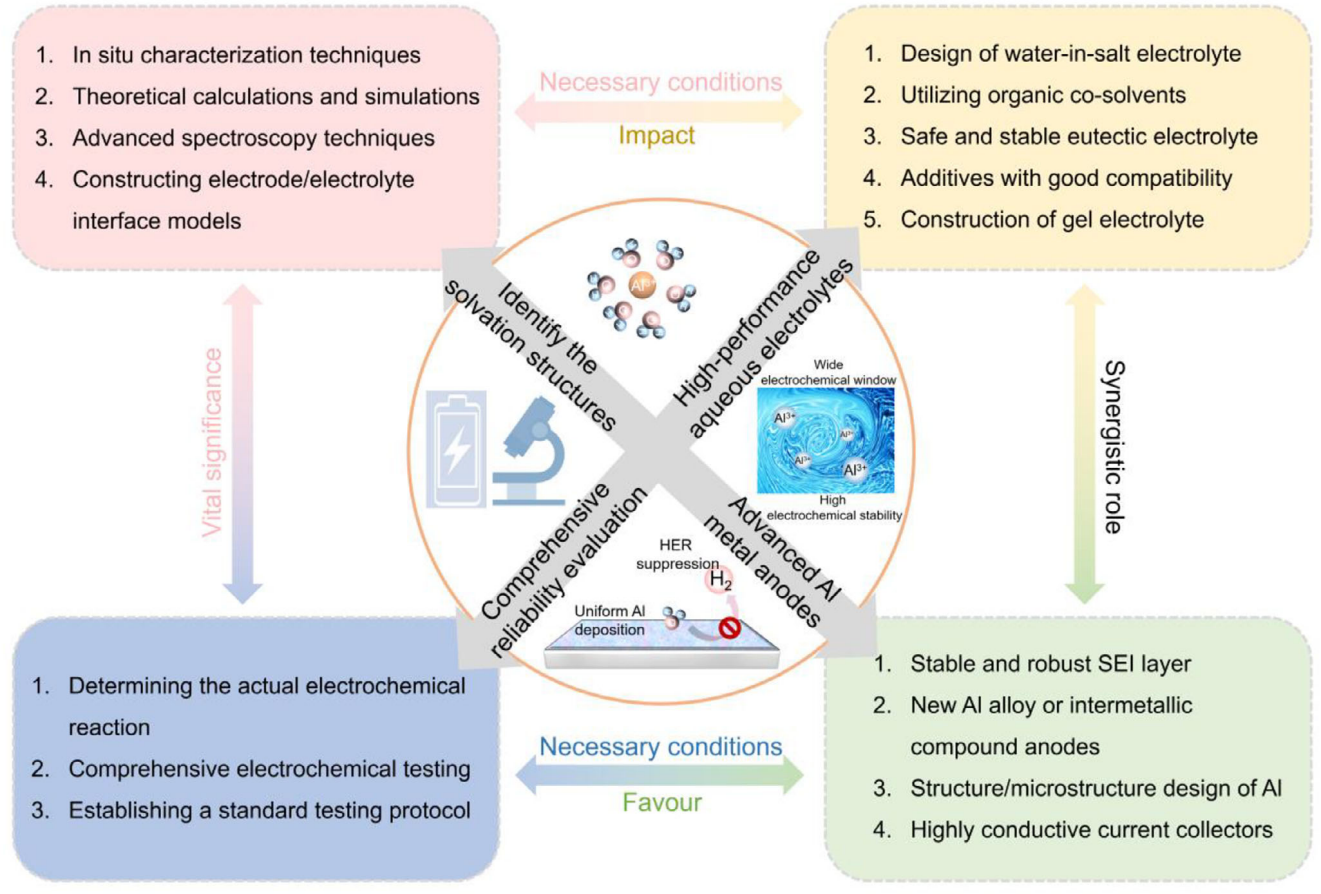
Perspectives of developing high‐performance AAMBs.

AAMBs have fascinating application prospects in large‐scale ESS due to their advantages of high safety, high energy density, and low cost. Compared with non‐aqueous aluminum ion batteries and other aqueous batteries, research on AAMBs is still in its infancy and faces more urgent challenges that need to be addressed. The dense oxide film spontaneously generated on the surface of aluminum metal in air prevents the reactivity of aluminum metal. Aluminum metal inevitably undergoes corrosion, surface passivation, and HER in aqueous electrolytes, resulting in irreversible deposition/dissolution of aluminum ions. Meanwhile, the inherent narrow ESW, complex side reactions, and strong corrosiveness of aqueous electrolytes further limit the performance of AAMBs. In addition, due to the inherent large size and high charge density of hydrated aluminum ions, AAMB currently lacks cathode materials with high specific capacity and cycling stability. Under the combined effect of these challenges, achieving high‐performance AAMBs still requires significant advancements and improvements in design and implementation. This review focuses on how to activate and stabilize Al metal anodes for high performance rechargeable AAMBs, and summarizes the research progress and design strategies of aluminum metal anodes and aqueous electrolytes. Excitingly, in recent years, many researchers have designed various clever solutions to address the problems faced by AAMBs, resulting in certain improvements in their performance. In the future, to truly manufacture chemical energy storage devices using aluminum metal and aqueous electrolytes, it is still necessary to systematically solve the fundamental problems and application‐related challenges faced by AAMBs. Based on the findings of this review, we propose the following discussions and suggestions to promote the development of high‐performance AAMBs (**Figure** **12**).

(1) It is certain that aluminum metal is an ideal anode material, yet it is challenging to utilize directly at this stage. As is well known, the high bandgap, high‐density passivation film that spontaneously forms on the surface of aluminum anodes leads to slow aluminum deposition/stripping kinetics, resulting in low operating voltage and low power density of AAMBs. Although the dynamics of aluminum ions can be improved by physically removing the passivation film, the active interface accelerates hydrogen production. Regarding the issue of surface oxide films, advanced surface treatment techniques such as plasma treatment, chemical etching, or laser ablation could be used to selectively remove or modify the surface oxide layer on aluminum anodes. This can create a more reactive surface without compromising the overall stability of the anode. Meanwhile, developing a long‐term stable SEI and aluminum metal anode is crucial. Establishing an artificial interfacial functional layer on the surface of aluminum metal anodes is considered a potential solution to the challenges faced by aluminum anodes. Treating aluminum foil with ionic liquid/eutectic electrolytes can form a stable SEI layer while removing the surface passivation film. However, recent research has found that this SEI, which provides the role of chlorine ion‐induced aluminum corrosion reactions, does not prevent the HER. Coating the surface of aluminum metal anodes with organic polymer layers can inhibit HER, but the repeated stripping/plating process during discharge/charging leads to interphase rupture and increases the migration barrier for aluminum ions. Therefore, constructing an inorganic/polymer hybrid SEI may be a possible strategy to address this issue, where the inorganic compounds provide good ionic conductivity for transporting Al^3^⁺, and the elastic polymers can accommodate the volume changes during the aluminum stripping/plating process. Meanwhile, advanced thin‐film technologies, such as chemical vapor deposition (CVD), inkjet printing, and Langmuir‐Blodgett techniques, are recommended to precisely control the uniformity, thickness, and defects of the thin films. Although strategies for constructing SEI on metal anodes have been widely studied in various metal battery systems, their reliability in AAMBs has always been questioned. In the future, this issue still requires in‐depth investigation and analysis. Using aluminum alloy or intermetallic compound anodes with antioxidants, high conductivity, and aluminophilicity is also an important strategy. Aluminum alloy anodes have higher discharge platforms and smaller polarization voltages. Designing multicomponent novel aluminum alloys through in situ eutectic solidification reactions and electrochemical reactions is conducive to obtaining more stable aluminum alloy anodes. In the design process of alloy anodes, it is recommended to use artificial intelligence and theoretical calculations to predict and select alloy elements with high corrosion resistance, high ionic diffusion coefficients, and high conductivity. In addition to constructing SEI and designing alloy anodes, designing the morphology and structure of aluminum metal can also achieve lower nucleation energy barriers and faster interfacial ion transfer kinetics.

(2) An ideal electrolyte should possess high ionic conductivity, high chemical stability, a broad ESW, and promote efficient and reversible aluminum plating/stripping while maintaining good interfacial stability. In AAMBs, the use of water as a solvent ensures the safety and low cost of the battery. Compared to traditional organic solvents and ionic liquid electrolytes, aqueous electrolytes exhibit lower charge transfer resistance and lower desolvation energy, which endows AAMBs with higher capacity and lower overpotential. It is worth noting that the use of different types and concentrations of aluminum salts significantly alters the electrolyte environment (pH, ionic conductivity, and viscosity, etc.), which greatly affects the migration and insertion of protons and aluminum ions. Furthermore, most current reports on AAMBs use organic aluminum salts (such as Al(OTF)_3_ or Al(ClO_4_)_3_) at varying concentrations, which significantly diminishes the cost advantage and environmental friendliness of aqueous electrolytes. Future research should focus on the development and utilization of other low‐cost and highly safe inorganic aluminum salts.

Although aqueous solvents have fascinating intrinsic advantages, complex interfacial reactions and severe corrosion of aluminum metal lead to unsatisfactory performance of AAMBs. In this context, the use of high‐concentration aluminum salts or water/organic mixed electrolytes may exhibit better electrochemical performance than in pure water. Recently reported HEEs are also highly attractive. In HEEs, water molecules exist as bound water and are coordinated with metal cations to remain associated. Due to the hydration effect, HEEs have relatively low viscosity, high ionic conductivity, and lower dissociation energy of Al^3+^ complexes. The solvation structure of aluminum ions directly affects the stability of AEI and the deposition/dissolution of aluminum. By altering the solvation structure of aluminum ions, surrounding the Al^3^⁺ in the electrolyte with a high concentration of anions rather than water molecules, this can preferentially suppress HER and interfacial corrosion. Furthermore, by reducing the desolvation barrier of Al^3+^ complexes, preferential deposition of aluminum ions can be achieved, thus fully utilizing the high theoretical specific capacity brought by the three‐electron transfer reaction.

The desolvation energy of Al^3+^ and its solvation structure can also be adjusted by modifying the electrolyte composition and using additives. In the selection process of additives, attention should be paid to the mechanism of reaction, cost, electrode interface compatibility, and stability. Noticeably, the development of new electrolytes is a complex task, involving the selection and combination of aluminum salts, solvents, and organic ligands. In future exploration, it is recommended to combine existing experimental data, theoretical simulations, and advanced computer technologies (such as artificial intelligence and machine learning). By leveraging existing artificial intelligence models and machine learning techniques, the dissolution, complexation, and interaction of molecules in the electrolyte can be simulated to screen and optimize anions, solvents, and additives, and to optimize/predict the best electrolyte formulation for AAMBs, which can reduce the time consumption of experimental exploration. In the future, the synergistic use of stable aluminum metal anodes and new electrolytes may further harness the intrinsic advantages of AAMBs.

(3) In recent years, researchers have employed a variety of strategies to activate and stabilize aluminum metal anodes for improved electrochemical performance. However, the chemical state of Al^3+^ in the electrolyte remains unclear, with a lack of fundamental research, particularly concerning super‐concentrated electrolytes and mixed electrolytes. The types of aluminum salts used in AAMBs are numerous, encompassing a variety of anions. Different anions and concentrations of aluminum salts significantly affect the solvation structure of aluminum ions, making the solvation structure of Al^3+^ highly complex. For instance, in commonly used aluminum chloride electrolytes, there exist active ions such as AlCl_4_^−^, Al_2_Cl_7_^−^, Cl^−^, Al^3+,^ and H^+^. It is well known that under the presence of an electric field, desolvated water molecules can easily migrate to the anode and induce the HER process. Therefore, elucidating the solvation structure and chemical state of charge carriers in aqueous electrolytes is crucial for clarifying the corresponding electrochemical mechanisms. First, various characterization techniques and theoretical calculations should be utilized to explore the specific active components of the electrolyte, which is beneficial for determining the redox mechanisms and energy storage mechanisms at the aluminum anode interface. Advanced characterization techniques, such as nuclear magnetic resonance spectroscopy, inelastic neutron scattering, X‐ray absorption spectroscopy (XAS), and in situ FTIR/Raman spectroscopy, can provide insights into the solvation structure and desolvation processes of Al^3+^ ions. Additionally, systematic density functional theory (DFT) calculations and molecular dynamics (MD) simulations can be combined with characterization techniques and further verify the reliability of the results. DFT calculations can provide information on the forces between ions and molecules, offering theoretical predictions for preliminary judgments of the chemical state of aluminum ions. Moreover, DFT calculations can analyze the energy values required for aluminum ions to form or separate from the solvation structure, calculate the diffusion energy barriers and pathways for charge carriers at the interface, which helps to reveal the ion diffusion mechanisms and screen components that favor reducing the desolvation energy barrier of aluminum ions. Concurrently, MD simulations can directly obtain the solvation structure of aluminum ions in the electrolyte and their dynamic changes under the influence of an electric field. It can also provide data on the formation of the double layer and changes in hydrogen bonds within the electrolyte, which aids in comprehensively understanding and analyzing the existence state of aluminum ions in the electrolyte system and the dynamic changes of other components.

(4) Currently, there is still no definitive conclusion regarding whether aluminum ions can reversibly deposit on the aluminum metal anode in aqueous solutions. Some researchers argue that there is no reversible aluminum deposition, but rather an illusion caused by the continuous dissolution of the aluminum anode and hydrogen evolution. Moreover, the complex solvation structures and the presence of various ions in the electrolytes of AAMBs further complicate the study of the electrochemical deposition mechanism of aluminum. It is necessary to combine advanced characterization techniques with theoretical calculations to elucidate the different reaction mechanisms on the AEI when using different types of electrolytes, thereby providing fundamental principles for the future design of AAMBs. When analyzing and verifying the deposition behavior of Al^3+^, the low amount of aluminum deposition and the rapid passivation of aluminum make it difficult to obtain accurate surface morphology and composition results using ex‐situ characterization techniques. Therefore, in situ optical microscopy/scanning electron microscopy (SEM)/ transmission electron microscopy (TEM)/X‐ray diffraction (XRD)/electrochemical quartz crystal microbalance (EQCM) could be employed to accurately analyze the aluminum deposition process and monitor the complex interfacial reactions occurring on the aluminum metal anode. Furthermore, the testing and evaluation of electrochemical performance of AAMBs are currently quite inconsistent. The stability of Al metal anode is often tested under the conditions of coin cells or even three‐electrode systems, with low applied current densities (0.05–0.5 mA cm^−2^) and low areal capacities. This leads to the neglect of most side reactions and makes it difficult to verify the true performance of modified aluminum anodes and electrolytes, and even fails to rule out the interference of continuous hydrogen evolution rather than aluminum ion deposition. Additionally, the main issues that may arise during the long‐term use of the batteries include capacity fading, decreased cycle stability, and increased internal resistance. These problems mainly stem from the instability of aluminum metal anodes in aqueous solutions, such as self‐corrosion, surface passivation, or hydrogen evolution reactions. These side reactions significantly reduce the reaction activity, hinder the reversible deposition/dissolution of aluminum, and limit the electrochemical performance of AAMBs. Therefore, during the evaluation and testing process, special attention should be paid to high current densities, high depth of discharge, high cathode loading, lean electrolyte, long cycle time, and large cell volume (high overall capacity) to verify whether the designed battery can fully leverage the advantages of the aluminum metal anode and is suitable for commercial applications.

In summary, AAMBs are a promising candidate in the field of next‐generation large‐scale ESS, but issues such as passivation of aluminum anodes and difficulty in achieving reversible aluminum deposition have long plagued researchers. In the past decade, research on AAMBs has been significantly less than that on organic battery systems and even other aqueous metal batteries. However, due to the outstanding inherent advantages of aluminum metal, AAMBs still have high research value. Despite some strategies may resonate with other aqueous batteries in the design process of new aluminum metal anodes and aqueous electrolytes, tailoring exclusive solutions is crucial due to the unique ionic properties and interface reaction mechanisms of AAMBs. Although AAMBs still face significant challenges at present, we believe that as the demand for efficient and sustainable batteries grows and the development of various components in AAMBs progresses, they will meet the future requirements for low‐cost, high‐safety, and high‐performance ESS.

## Conflict of Interest

The authors declare no conflict of interest.
